# Spatio-chromatic information available from different neural layers via Gaussianization

**DOI:** 10.1186/s13408-020-00095-8

**Published:** 2020-11-11

**Authors:** Jesús Malo

**Affiliations:** Image Processing Lab, Universitat de València, Catedrático Escardino, 46980 Valencia, Paterna Spain

**Keywords:** Retina–cortex pathway, Cones, Chromatic adaptation, Opponent channels, Chromatic saturation, Texture sensors, Chromatic and achromatic Contrast sensitivity functions (CSFs), Divisive normalization, Transmitted information, Total correlation, Mutual information, Gaussianization

## Abstract

*How much visual information about the retinal images can be extracted from the different layers of the visual pathway?* This question depends on the complexity of the visual input, the set of transforms applied to this multivariate input, and the noise of the sensors in the considered layer. Separate subsystems (e.g. opponent channels, spatial filters, nonlinearities of the texture sensors) have been suggested to be organized for optimal information transmission. However, the efficiency of these different layers has not been measured when they operate together on colorimetrically calibrated natural images and using multivariate information-theoretic units over the joint spatio-chromatic array of responses.

In this work, we present a statistical tool to address this question in an appropriate (multivariate) way. Specifically, we propose an empirical estimate of the information transmitted by the system based on a recent Gaussianization technique. The total correlation measured using the proposed estimator is consistent with predictions based on the analytical Jacobian of a standard spatio-chromatic model of the retina–cortex pathway. If the noise at certain representation is proportional to the dynamic range of the response, and one assumes sensors of equivalent noise level, then transmitted information shows the following trends: (1) progressively deeper representations are better in terms of the amount of captured information, (2) the transmitted information up to the cortical representation follows the probability of natural scenes over the chromatic and achromatic dimensions of the stimulus space, (3) the contribution of spatial transforms to capture visual information is substantially greater than the contribution of chromatic transforms, and (4) nonlinearities of the responses contribute substantially to the transmitted information but less than the linear transforms.

## Introduction

Neuroscience has a long tradition in using information theory both to quantify the performance of neurons [1] and to formulate principles that explain observed behavior using the so-called *Efficient Coding Hypothesis* [2, 3]. Information theory is useful at many scales of sensory processing [4–6].

On the one hand, a large body of the literature studies information transmission starting from a spiking neuron [7], including all sorts of additional constraints such as energy or size [8–12]. From these estimates of transmitted information in single cells, different summation strategies [13] or independence assumptions [14] are considered to give global estimates of the amount of information transmitted by a set of sensors.

This detailed low-level descriptions are certainly the basis for higher-level behavioral effects, but psychophysics is usually described with more abstract models. In fact, cascades of *linear*+*nonlinear* layers of more schematic neurons [15–18] describe a wide range of psychophysical phenomena, including color [19, 20], spatial texture [21, 22], and motion [23]. On the other hand, these more schematic neural layers are also studied according to the *Efficient Coding Hypothesis*. Examples include the emergence of linear opponent chromatic channels [24] and their saturation [25–27], and the emergence of linear texture sensors (not only achromatic [28, 29], but also spatio-chromatic [30, 31], equipped with chromatic adaptation [32]). Here texture sensors refer to mechanisms with receptive fields with excitatory and inhibitory patterns of different frequency and orientation. Redundancy reduction also explains the emergence of linear sensors sensitive to motion [33]. Finally, the saturation of the responses of these spatio-temporal sensors has also been derived from information theoretic arguments in the case of achromatic textures [34, 35] and motion [36].

In this work, we use these higher-level models [18–23], which, being connected to physiology, are more related to the psychophysics of color and spatial texture. Information-theoretic study of psychophysical models may address biologically relevant questions like: *What perceptual behavior is more relevant to encode images? color constancy? or contrast adaptation?* *What mechanisms contribute to extract more information about the images? the chromatic opponent channels or the texture filters?* *What is the improvement due to the nonlinearities? being more flexible is always better?* *What kind of images are better represented by the visual system? smooth achromatic shapes or sharp chromatic patterns?*

Quantitative answers to these questions require reliable estimators of *transmitted information* over the cascade of layers. These estimators have to work on high-dimensional vectors, and more importantly, estimations should not depend on the specific expression of the response so that they could be applied to any model or even to raw experimental data.

In this work, we introduce a recent estimator of *mutual information* between multivariate variables based on Gaussianization [37–39] to address relevant sensory questions such as those stated before. Here we illustrate the kind of answers that can be found through this method by exploring the transmission of spatio-chromatic information through a cascade of standard linear+nonlinear layers that address in turn basic psychophysical phenomena on color and texture [19–22]. A model of known analytic Jacobian [40, 41] is convenient because, for some redundancy measures such as *total correlation*, the model-free estimations proposed here can be compared to estimations that use the analytical model. It is interesting that here we discuss that the analytical insight obtained from the model Jacobian for *total correlation* (as done in [40, 42, 43]) is not always applicable to predict the *transmitted information*. However, this poses no major problem if the estimator at hand does not depend on the model, which is the case in the proposed method.

The conventional way to check the Efficient Coding Hypothesis goes in the *statistics-to-biology* direction. In that case, biologically plausible behaviors are shown to emerge from the optimization of specific information-theoretic measures [24–36]. On the contrary, following [43, 44], here we go in the opposite direction from *biology to statistics*. Here we take a standard psychophysical model, which was not explicitly optimized to encode natural images, and we show it is remarkably good in terms of *transmitted information*.

Our results should not be misunderstood as a claim of *infomax* as the ultimate organization goal. Note that we do not propose to optimize the system according to that goal, but just show that a fixed plausible system performs quite well according to the goal. In fact, a large body of the literature includes additional constraints to information maximization [8–12, 26, 27, 29, 32, 36, 45]. Beyond disputes among specific goals, the method proposed here can be used to include *transmitted information* together with other goals in a combined cost function if the *statistics-to-biology* strategy is preferred.

## Materials: illustrative vision model and calibrated images

The Gaussianization tool presented in this work to measure the transmitted information is illustrated in a standard spatio-chromatic retina–cortex model fed with color-calibrated natural stimuli.

In this section, we first review the elements of this standard model, and then we show the distribution of natural images used in our experiments in terms of luminance, achromatic contrast, and chromatic contrast.

### A standard spatio-chromatic psychophysical pathway

The model considered here for illustrative purposes is a cascade of *linear*+*nonlinear* layers [18, 40, 41, 43]. In this setting, the *i*th layer takes the array of responses coming from a previous layer $\boldsymbol{x}^{(i-1)}$, applies a set of linear receptive fields that lead to the responses $\boldsymbol{r}^{(i)}$, and these outputs interact to lead to the saturated responses $\boldsymbol{x}^{(i)}$: 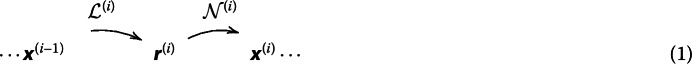


Specifically, the model used in the simulations below consist of three of such differentiable and invertible *linear*+*nonlinear* layers: 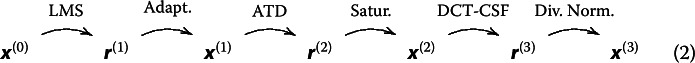 where we can identify the following standard processing elements (explicit equations in Appendix A).

#### Linear spectral integration

The spectral image $\boldsymbol{x}^{(0)}$ is analyzed at each spatial location by linear photoreceptors tuned to *long*, *medium,* and *short* (LMS) wavelengths; in particular, we use the standard cone fundamentals LMS in [46]. The $\boldsymbol{r}^{(1)}$ array contains the LMS retinal images.

#### Chromatic adaptation

We use the simplest chromatic adaptation scheme: the classical von Kries normalization [20], where the linear LMS signals are divided by an estimate of what is considered to be white in the scene. The $\boldsymbol{x}^{(1)}$ array contains the *von Kries-balanced* LMS retinal images.

#### Linear opponent color space

The LMS signals at each spatial location are linearly recombined into an *opponent* representation with Achromatic (luminance), Tritanopic (red–green) and Deuteranopic (yellow–blue) sensors. Specifically, $\boldsymbol{r}^{(2)}$ contains the achromatic, tritanopic, and deuteranopic (ATD) images of Jameson & Hurvich opponent sensors [47–49].

#### Weber-like saturation

The linear ATD responses saturate [50, 51] to give brightness (nonlinear A) and nonlinear versions of images T and D. This saturation can be modeled in sophisticated ways with psychophysical [20, 52, 53] or statistical grounds [26, 27], but in $\boldsymbol{x}^{(2)}$, we will use a simple dimensionwise nonlinearity using a $\gamma <1$ exponent with parabolic correction at the origin [40] to avoid singularities in the Jacobian. This exponential with fixed *γ* is the simplest model for the Weber-like luminance–brightness relation and the observed saturation in chromatic opponent channels [49].

Up to $\boldsymbol{x}^{(2)}$ the model consists of purely chromatic transforms that operate at each spatial location. In these initial layers, spatial context is not considered except for the scarce use made in chromatic adaptation to estimate the *white*. The final *linear*+*nonlinear* layer addresses spatio-chromatic texture.

#### Linear texture filters and contrast sensitivity

In the simple model considered here, spatial transforms are applied over all images A, T, and D in parallel. Neglecting the interactions between ATD images is consistent with the results found in analyzing the spatio-chromatic statistics of natural images: the chromatic variation of the statistical filters follows von Kries-corrected ATD directions regardless of the spatial distribution of the receptive field [32]. Here we use a crude local-DCT model for the local-oriented receptive fields in V1: the local oscillations applied to channel A account for the achromatic texture sensors [54], and those applied to arrays *T* and *D* account for the double-opponent cells [55]. These texture sensors are shown in Appendix A. The gain of these linear filters (receptive fields) is weighted according to their frequency using achromatic and chromatic contrast sensitivity functions (CSFs) [56, 57]. The weights for the local-DCT functions are based on the CSFs of the standard spatial observer defined for sinusoids [58] and on a procedure to transfer the weights from one domain to another [59]. The bandwidths of the achromatic and chromatic CSFs are markedly different. This bandwidth is related to the frequency response of magno, parvo, and konio cells [55, 60, 61]. The array $\boldsymbol{r}^{(3)}$ consists of the spatial transforms of patches A, T, and D, frequency weighted and stacked one after the other.

#### Nonlinear interactions between texture sensors

Following [15–18, 22], the saturation of the sensors tuned to chromatic/achromatic textures has been modeled using a psychophysically-tuned divisive normalization [40, 41, 62]. Note that alternative cortical nonlinearities such as Wilson–Cowan equations [63] have been found to be equivalent to divisive normalization [64]. The same parameters for the normalization have been used for the achromatic part and the chromatic parts of the response. As in the linear case, no interaction has been considered between channels A, T, and D . Appendix A illustrates the impact of the parameters of divisive normalization on the flexibility of the nonlinearity.

### Plausibility of the psychophysical model

Here we illustrate the plausibility of the specific blocks described before (with specific parameters described in Appendix A) by predicting results in image distortion psychophysics.

In image quality databases [65], observers assess the visibility of a range of distortions seen on top of natural images. A vision model is good if the predicted visibility correlates with human opinion. The visibility of a distortion from a psychophysical response model is computed by measuring the distance from the response vector of the original image to the response vector of the distorted image [66, 67]. In this context, we made two simple numerical experiments: (1) we checked if the consideration of more and more standard blocks in the model leads to consistent improvements of the correlation with human opinion, and (2) we checked how substantial modifications to the standard blocks affect its performance. In particular, (a) we changed the flexibility of the baseline masking model by decreasing or increasing the semisaturation constant in the denominator of the divisive normalization, which leads to more flexible and more rigid models, respectively; and (b) we generated a totally rigid model by neglecting all the nonlinearities. Appendix A illustrates these changes in flexibility of the response.

**Figure 1:**
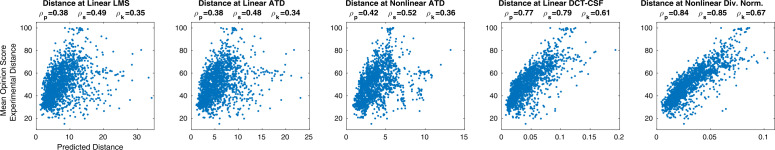
Correlation with human opinion as additional layers are added to the model. As different illuminations are not present in the considered database [65], we cannot assess the effect of the von Kries normalization on LMS (missing intermediate step between linear LMS and linear ATD)

Figure 1 confirms that the progressive consideration of the blocks leads to consistent improvements in correlation. The final model (the whole cascade of standard modules) is plausible because of two reasons. First, the final correlation with human behavior is similar to state-of-the-art image quality metrics [40, 41, 58, 68]. Second, Table 1 shows that the cascade of standard modules (baseline model) is better than more adaptive or more rigid versions of the model. This means that the considered parameters make biological sense. Of course, the correlation substantially increases with the spatial size of the image patches and receptive fields, but a model with *exactly the same parameters* on small image patches shows the same trend when considering the series of layers. These results illustrate the meaningfulness of the considered psychophysical blocks and its appropriate behavior when functioning together.

**Table 1:** Pearson correlation with human viewers using different building blocks (or model layers)

	Spatial extent	$\boldsymbol {r}^{(1)}$	$\boldsymbol {r}^{(2)}$	$\boldsymbol {x}^{(2)}$	$\boldsymbol {r}^{(3)}$	$\boldsymbol {x}^{(3)}$
More flexible model	(0.27 deg)	0.38	0.38	0.42	0.77	0.51
**Baseline model**	(0.27 deg)	0.38	0.38	0.42	0.77	**0.84**
More rigid model	(0.27 deg)	0.38	0.38	0.42	0.77	0.79
Totally rigid model	(0.27 deg)	0.38	0.38	0.38	0.68	0.68
Baseline model	(0.05 deg)	0.26	0.27	0.31	0.37	0.40

Finally, intuition about the information-theoretic performance of the considered model may be obtained by visualizing the geometrical effect of the series of transforms on the manifold of natural images. This intuition is discussed in Appendix B.

### Calibrated natural images

**Figure 2:**
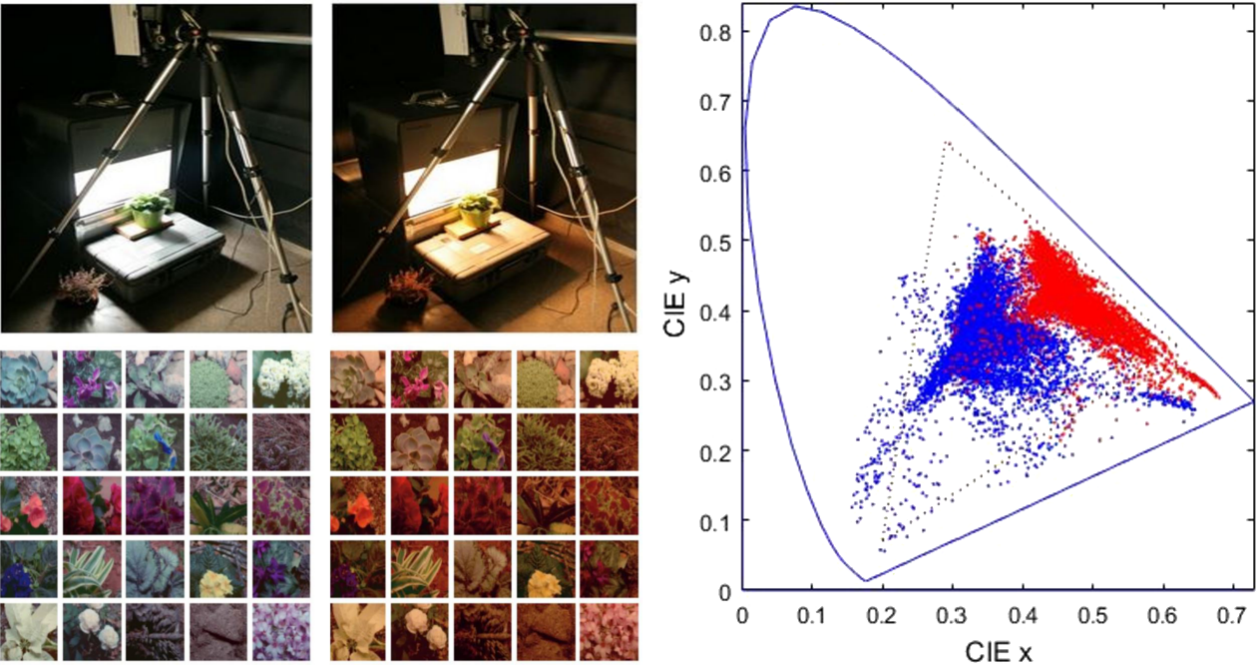
Calibrated images used in the experiments: natural scenes under CIE D65 (left image panel) and CIE A (right image panel) illuminations. The CIE *xy* diagram shows the shift in chromaticity (samples under D65 in blue and samples under A in red)

The IPL color image database [27, 32, 36] is well suited to study color adaptation because its controlled illumination under CIE A and CIE D65 allows straightforward application of von Kries adaptation. With the knowledge of the illumination, there is no need for extra approximations (e.g. gray-world) to estimate the white. Controlled scene acquisition and resulting CIE *xy* data are illustrated in Fig. 2.

Alternative calibrated choices could be the spectral-image datasets [69, 70] or the tristimulus-calibrated dataset [71], which also provide the illumination information from gray spheres located in the scenes.

We extracted $19\cdot 10^{6}$ image patches from the database (expressed in CIE XYZ tristimulus values) and transformed them into the linear LMS representation. In fact, in this work, we consider the behavior of the model from $\boldsymbol{r}^{(1)}$. This amounts to considering that the input to the system is the set of linear LMS images. To keep the dimensionality small for a proper comparison of the empirical and theoretical estimates of information, we kept the spatial extent small, only $3\times 3$ pixels. As a result, the input stimuli $\boldsymbol{r}^{(1)}$ and the response arrays live in 27-dimensional spaces.

To check whether the *transmitted information* through the system is adapted to the statistics of the natural input, we consider the distribution of the considered samples over three relevant visual features: the average luminance, the achromatic contrast, and the chromatic contrast of the pattern in the image. To define these features, LMS images are expressed in the Jameson & Hurvich ATD space using no chromatic adaptation. In this representation, average luminance in cd/m^2^ units is simply the average of image A. The achromatic contrast is defined as the RMSE contrast of image A (standard deviation divided by the mean luminance). Similarly, the chromatic contrast is defined as the mean of the RMSE contrasts of images T and D, where the corresponding standard deviation is divided by the norm of the average color in the patch.

**Figure 3:**
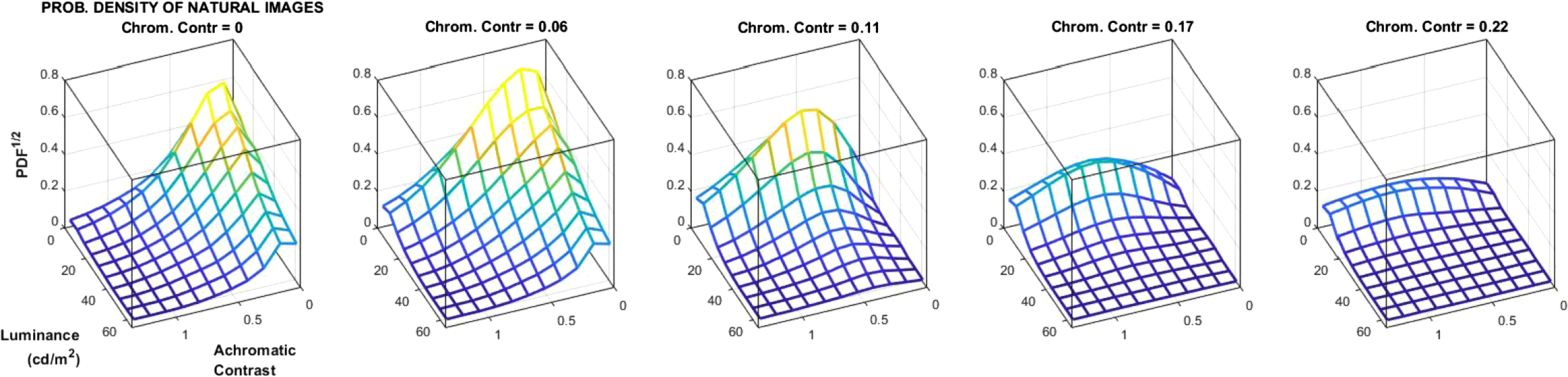
Probability of natural scenes as a function of *chromatic contrast*, *achromatic contrast*, and *luminance*: dark and smooth patches are more frequent

By computing these three features for the $19 \cdot 10^{6}$ images we find a markedly nonuniform probability density function (PDF): image patches are mostly dark, and the variance of the deviations with regard to the mean is small both in luminance and in color (see Fig. 3). This distribution shows that in nature some regions of the image space are more frequent that others. The efficient coding hypothesis generically argues that the performance of the visual system should be shaped by this uneven distribution. Here we propose a quantitative comparison. In Sect. 5, we plot the information transmitted up to the cortical sensors (at layer $\boldsymbol{x}^{(3)}$ in Eq. (2)) over the luminance/contrast dimensions to check if the transmitted information for specific images is related to how frequent these images are.

## Methods: transmitted information from Gaussianization

In a sensory system where the input undergoes certain deterministic transform *S*, but the sensors are noisy: 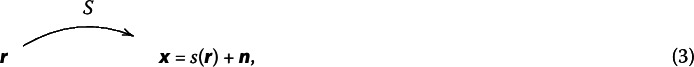 the mutual information between input and output, $I(\boldsymbol{r},\boldsymbol{x})$, is the information about the input vector ***r***, which is shared by the response vector ***x*** [72] (Chap. 2). We will refer to $I(\boldsymbol{r},\boldsymbol{x})$ as the information about the input captured by the response or as the information transmitted from the input to the response. This mutual information $I(\boldsymbol{r},\boldsymbol{x})$ is also the information about the input available at the representation ***x***.

In the context of the *Efficient Coding Hypothesis*, it is interesting to compare different image representations $s(\boldsymbol{r})$ in terms of the amount of information they capture about the input for some given noise ***n***. This would tell us about the relative contribution of the different operations in a model to get a better representation of the visual signal. Ideally, we would like to describe the trends of this performance measure $I(\boldsymbol{r},\boldsymbol{x})$ from the analytical description of the response $s(\boldsymbol{r})$, but this may not be possible, as discussed later in Sect. 6. Therefore empirical estimations from stimulus-response pairs are very interesting.

Estimation of $I(\boldsymbol{r},\boldsymbol{x})$ directly from samples and using the definitions based on the PDFs is not straightforward: it implies the estimation of multivariate PDFs, and this challenging problem introduces substantial bias in the results. This is also true for surrogates for performance such as the reduction of redundancy measured by the reduction in *total correlation* $\Delta T(\boldsymbol{r},\boldsymbol{x})$ [42].

In this work, we solve the estimation of the transmitted information up to different layers of the visual pathway through the relation between the *transmitted information* *I* and the *total correlation* *T*. In particular, we use a novel estimator of *T* [38, 39], which only relies on (easy-to-compute) univariate density estimations: the rotation-based iterative Gaussianization (RBIG) [37].

The RBIG is a cascade of *L* *nonlinear*+*linear* layers, and the *l*th layer is made of marginal Gaussianizations $\Psi ^{(l)}(\boldsymbol{x}^{(l)})$, followed by a rotation $R^{(l)}$. Each of such layers is applied to the output of the previous layer:

4 $$ \boldsymbol{x}^{(l+1)} = R^{(l)} \cdot \Psi ^{(l)}\bigl(\boldsymbol{x}^{(l)}\bigr). $$

For a big enough number of layers, this invertible architecture is able to transform any input PDF $p(\boldsymbol{x}^{(0)})$ into a zero-mean unit-covariance multivariate Gaussian even if the chosen rotations are random [37]. Theoretical convergence is obtained as the number of layers tends to infinity. However, in practical situations, early stopping criteria can be proposed taking into account the uncertainty associated with a finite number of samples [37]. Convergence even with random rotations implies that both elements of the transform are straightforward, univariate equalizations and random rotations. The differentiable and invertible nature of RBIG makes it a member of the normalizing flow family [73, 74]. Within this general family, differentiable transforms with the ability to remove all the structure of the PDF of the input data are referred to as *density destructors* [75]. By density destruction the authors in [75] mean a transform of the input PDF into a unit-covariance Gaussian or into a *d*-cube aligned with the axes. The considered Gaussianization [37, 38] belongs to this family by definition. *Total correlation* describes the redundancy within a vector, that is, the information shared by the univariate variables [76, 77]. Note that strong relations between variables indicate a rich structure in the data.

Density destruction together with differentiability is useful to estimate the total correlation within a vector $T(\boldsymbol{x}^{(0)})$. Imagine that the considered RBIG transforms the PDF of the input $\boldsymbol{x}^{(0)}$ into a Gaussian through the application of *L* layers (*L* individual transforms). As the redundancy of the Gaussianized signal $g_{\boldsymbol{x}}(\boldsymbol{x}^{(0)}) = \boldsymbol{x}^{(L)}$ is zero, the redundancy of the original signal $T(\boldsymbol{x}^{(0)})$ should correspond to the cumulative sum of the individual variations $\Delta T^{(l)}$ with $l=1, \ldots ,L$, which take place along the *L* layers of RBIG while converting the original variable ***x*** into the Gaussianized variable $g_{\boldsymbol{x}}(\boldsymbol{x})$. It is interesting that the individual variation in each RBIG layer only depends on (easy to compute) univariate negentropies, and therefore, after the *L* layers of RBIG, the total correlation is [39]

5 $$ T(\boldsymbol{x}) = \sum_{l = 1}^{L} \Delta T\bigl(\boldsymbol{x}^{(l-1)}, \boldsymbol{x}^{(l)} \bigr) = \sum_{l=1}^{L} J_{m}\bigl(\boldsymbol{x}^{(l)}\bigr), $$

where the marginal negentropy of a *d*-dimensional random vector is given by a set of *d* univariate divergences $J_{m}(\boldsymbol{v}) = \sum_{i=1}^{d} D_{\mathrm{KL}}(p(v_{i})| \mathcal{N}(0,1))$. Therefore, using RBIG, the challenging problem of estimating one *d*-dimensional joint PDF to compute $T(\boldsymbol{x})$ reduces to solving $d\times L$ univariate problems. Moreover, as opposed to the computation of variations of total correlation depending on the model transform, as, for instance, Eq. (9) discussed in Sect. 6, RBIG estimation is model-free and does not involve any averaging over the whole dataset.

In the density destructor framework, where *T* is easy to compute using RBIG, the transmitted information from the input LMS image, ***r***, to any of the considered layers downstream ***x***, namely $I(\boldsymbol{r},\boldsymbol{x})$, can be obtained from *T* in two ways:

6 $$\begin{aligned} I(\boldsymbol{r},\boldsymbol{x}) =& T\bigl([\boldsymbol{r}, \boldsymbol{x}] \bigr) - T(\boldsymbol{r}) - T(\boldsymbol{x}), \end{aligned}$$

7 $$\begin{aligned} I(\boldsymbol{r},\boldsymbol{x}) =& T\bigl(\bigl[g_{\boldsymbol{r}}( \boldsymbol{r}),g_{\boldsymbol{x}}(\boldsymbol{x})\bigr]\bigr), \end{aligned}$$

where the relation in Eq. (6) is straightforward from the definitions of *I* and *T* in terms of entropies, and the sum would imply three Gaussianization transforms: one for the input, one for the responses, and an additional one for the concatenated input-response vectors $[\boldsymbol{r}, \boldsymbol{x}]$. Equation (7) also implies three Gaussianization transforms: the variables $g_{\boldsymbol{r}}$ and $g_{\boldsymbol{x}}$ come from the Gaussianization transforms of the images and the neural responses, respectively, and then we make a single computation of total correlation for the concatenated variable $[g_{\boldsymbol{r}}(\boldsymbol{r}),g_{\boldsymbol{x}}( \boldsymbol{x})]$ through an extra Gaussianization. It is important to note that, as opposed to Eq. (6), the strategy represented by Eq. (7) only implies *one* computation of *T*, not three.

Equation (7) is possible because *I* does not change under invertible transformations applied separately to each dataset [78]. Therefore $I(\boldsymbol{r},\boldsymbol{x}) = I(g_{\boldsymbol{r}}( \boldsymbol{r}),g_{\boldsymbol{x}}(\boldsymbol{x}))$. Since we removed *T* within each individual dataset by applying individual density destructors, the only redundant information that remains in the concatenated vectors is that shared by the original datasets, and therefore $I(g_{\boldsymbol{r}}(\boldsymbol{r}),g_{\boldsymbol{x}}( \boldsymbol{x})) = T([g_{\boldsymbol{r}}(\boldsymbol{r}),g_{ \boldsymbol{x}}(\boldsymbol{x})])$, and hence Eq. (7). See [39] for a more elaborate proof.

These two strategies (6) and (7) involve different numbers of computations of‘*T*. Therefore their accuracy depends on the nature of the data. Appendix C compares the estimations of transmitted information given by Eqs. (6) and (7) (and other estimators [79–81]) in scenarios where analytical solutions are available. The result of such an analysis is that for image-like heavy tailed variables, the sum in Eq. (6) leads to a greater error. Therefore results in the experimental section are computed using Eq. (7).

## Experiments

We conduct experiments to quantify (1) how much visual information is captured by different image representations along the layers of the neural model and (2) how much information is transmitted by the vision model for images with different visual features (e.g. different *luminance*, *achromatic contrast*, or *chromatic contrast* as considered in Sect. 2.3).

Here we describe the magnitudes measured. Then we describe the assumptions on the noise made throughout the experiments, and finally we describe *global* and *local* experiments, that is, made for *all* visual scenes and for scenes with *specific features*, respectively.

### Measurements of transmitted information and redundancy reduction

In the experiments, we measure the mutual information between the LMS input and different layers of the considered networks, $I(\boldsymbol{r}^{(1)},\boldsymbol{x})$, where ***x*** stands for one of the considered image representations (or layers in Eq. (2)). Therefore $I(\boldsymbol{r}^{(1)},\boldsymbol{x})$ is the amount of information transmitted up to layer ***x*** or available at layer ***x***. We also check how the input redundancy at the retinal representation, measured in terms of *total correlation*, gets reduced along the different image representations, that is, we compute $\Delta T(\boldsymbol{r}^{(1)},\boldsymbol{x}) = T(\boldsymbol{r}^{(1)}) - T(\boldsymbol{x})$ for different layers ***x***.

The RBIG estimator of *I* uses Eq. (7). The RBIG estimator of Δ*T* uses Eq. (5) to estimate $T(\boldsymbol{r}^{(1)})$ and $T(\boldsymbol{x})$, and then it subtracts these results.

Regarding the ability to interpret the visual world, measuring the information available at certain image representation is more related to the visual function than measuring the (more technical) amount of redundancy reduced at that layer. Redundancy reduction is sometimes taken as a convenient surrogate of transmitted information, but, as discussed further in Sect. 6, Δ*T* and *I* are not always aligned. However, the analysis of Δ*T* is technically interesting because alternative estimators of Δ*T* based on the analytical expression of the vision model can be used to check the accuracy of the RBIG estimators. As the alternative estimator of Δ*T* discussed in Sect. 6, Eq. (9), only depends on the analytical expression of the model and on univariate entropies (for which very reliable estimators exist [80]), it will be referred to as *theoretical estimation*. The RBIG estimations of Δ*T* will be compared to this *theoretical estimation* given by Eq. (9), and checking Δ*T* is important because the proposed estimator of *I* relies on measures of *T*, as seen in Eqs. (6) and (7).

### Assumptions on the noise and interpretation of results

Transmitted information between two layers of a network depends on the noise in the response. The noise in psychophysical systems is related to the discrimination ability of the observers [82, 83], but the nature of the noise is still under debate [84, 85]. Noise may have different sources, and its amount may depend on attention [86–88].

The specific debate on the noise is out of the scope of this work. However, the advantage of the RBIG estimate of transmitted information is that it does not rely on a specific analytical PDF of the noise, but only on the availability of noisy samples. Therefore it can handle responses corrupted with arbitrary noise sources.

Our experiments to estimate *I* consider a crude noise model using the following assumptions.

- *Assumption 1: single-step transforms.* In the path from the input up to the *i*th layer, we assume that noise is added to the signal *only* at the *i*th layer. This choice is equivalent to assuming that the mapping into the *i*th representation is a *single-step transform* in which all the intermediate stages are noise-free.
- *Assumption 2: fixed signal-to-noise ratio (SNR).* The additive noise is assumed to be Gaussian with diagonal covariance. Moreover, each marginal standard deviation is assumed to be proportional to the mean amplitude of the signal for that coefficient at the *i*th layer. Specifically, we set the noise standard deviation at 5% of the amplitude of the signal in every case.

*Assumption 1* should be understood correctly to avoid misinterpretations of the results. For instance, considering $\boldsymbol {r}^{(1)} \rightarrow \boldsymbol {x}^{(2)}$ and $\boldsymbol {r}^{(1)} \rightarrow \boldsymbol {x}^{(3)}$ as *single-step transforms* means that noise is added in $\boldsymbol {x}^{(2)}$ in the first case and in $\boldsymbol {x}^{(3)}$, but not in $\boldsymbol {x}^{(2)}$, in the second case. There is no problem to compare the representations $\boldsymbol {x}^{(2)}$ and $\boldsymbol {x}^{(3)}$. Particularly, if the mechanisms that perform these *single-step transforms* have the same quality in terms of signal-to-noise ratio (set in *Assumption 2*). The design question under these assumptions is: if you were able to build a mechanism to lead you either to representation $\boldsymbol {x}^{(2)}$ or $\boldsymbol {x}^{(3)}$, but the quality of the mechanism could not be better than certain SNR, which image representation would you prefer?

On the contrary, assuming that $\boldsymbol {r}^{(1)} \rightarrow \boldsymbol {x}^{(3)} \equiv \boldsymbol {r}^{(1)} \rightarrow \boldsymbol {x}^{(2)} \rightarrow \boldsymbol {x}^{(3)}$, where noise is injected *both* in $\boldsymbol {x}^{(2)}$ *and* in $\boldsymbol {x}^{(3)}$, is a quite different alternative scenario. Under *Assumption 1,* the information captured by $\boldsymbol {x}^{(3)}$ may be greater than the information captured by $\boldsymbol {x}^{(2)}$, but this is not be possible in the alternative scenario where noise is injected at every layer. In this latter case the *data processing inequality* [72] (Chap. 2) states that the information lost (due to the noise) at the second layer cannot be recovered afterward.

*Assumption 1* (or the *single-step transform* assumption) and the alternative *multiple-step transform* are just different ways to consider where the noise comes from. Given that no conclusive prescription for the amount of noise is available for *all* the considered psychophysical stages [82–88], arbitrary assumptions would also be necessary in the *multiple-step transform* case.

In summary, for illustration purposes, *Assumption 1* is valid as the alternative *multiple-stage transform* scenario, and it allows us to compare different image representations with a clear design restriction (fixed signal-to-noise ratio). Nevertheless, it is clear that results obtained from *Assumption 1* have not to be interpreted in terms of the data processing inequality. This is because in all the experiments, we compare multiple *single-step transforms* and not cascades of noisy blocks in which information loss propagates though the network. Similarly, the specific nature of the noise and the specific noise level set by *Assumption 2* are just convenient choices for illustration purposes.

To conclude, let us recall again that RBIG estimation does not depend on the noise model. Therefore the procedure described further would not change using different assumptions (i.e. more sophisticated uncertainties in the sensors or noisy responses from actual measurements).

### Global and local experiments

Information measures are defined as integrals over the whole space of inputs $\boldsymbol{r}^{(1)}$ and the corresponding responses ***x***. Experiments considering stimuli all across the input space will be referred to as *global*. Nevertheless, it is also interesting to consider how different regions of the image space are encoded. For instance, stimuli with specific visual features as those introduced in Sect. 2.3, for example, different *luminance*, *achromatic contrast*, or *chromatic contrast*. It is possible that a sensory system is specialized on stimuli with certain features. This question can be quantified by computing the amount of transmitted information about samples belonging to specific regions of the image space. Experiments considering specific regions of the image space will be referred to as *local*, that is, *local* in the space of image features.

In the *global* experiments below, 10 realizations of each estimation are done using $0.5 \cdot 10^{6}$ randomly chosen stimuli from the image dataset that consists of $19\cdot 10^{6}$ samples. Of course, the corresponding responses are also considered in each case.

In the *local* experiments below, the estimations at each location of the stimulus space are based on the images belonging to the corresponding luminance/contrast bin of the histogram shown in Fig. 3. In the *local* experiments, 10 realizations of each estimation are done for the data in each bin. In principle, each realization randomly selects 80% of the samples available in the bin. However, in the low-luminance/low-contrast bins the population is very large, and considering that many samples slows down the estimation. Therefore no more than $5\cdot 10^{5}$ of these randomly chosen samples were considered in each case. On the other hand, bins with less than 500 samples (in the high-luminance/high-contrast corner) were discarded in the estimation because results may not be reliable. In the results below, in those low-populated bins, we plot a constant value from the boundary of bins with population bigger than 500, but this value is arbitrary, and these regions are not considered in the discussion.

In all the experiments, images and the corresponding responses are 27-dimensional as stated in Sect. 2.3. The specific measurements shown are as follows.

- *Global experiments:*
  - *Global 1*: Redundancy reduction at different layers of different models.
  - *Global 2*: Information available at different layers of different models.
- *Local experiments:*
  - *Local 1*: Redundancy reduction in V1 for different kinds of stimuli.
  - *Local 2*: Redundancy reduction at different layers of the model and at different locations of the image space.
  - *Local 3*: Information available at different layers for different stimuli.
  - *Local 4*: Information available at V1 and the PDF of natural images.

## Results

### Global experiment 1: redundancy reduction at different layers of different models

**Figure 4:**
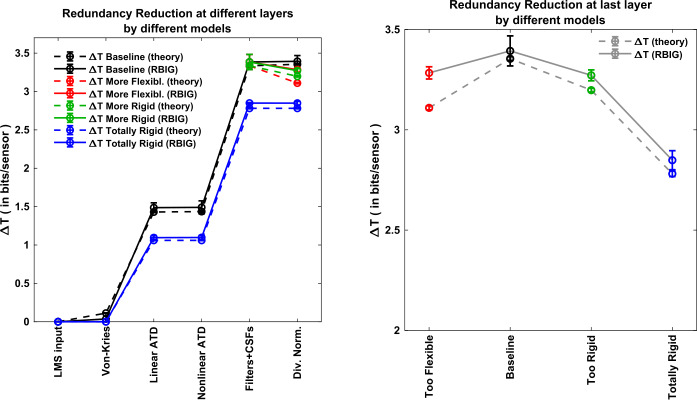
*Left*: Redundancy reduced at different layers of the network (labels in the horizontal axis) considering samples over the whole image manifold (*global* experiment). Redundancy is measured in total correlation (in bits/sensor). Lines in different colors refer to different models, and different line styles (solid/dashed) refer to the empirical RBIG estimate given by Eq. (5) and the *theoretical estimate* given by Eq. (9). Each result accumulates the reductions due to previous layers. *Right*: Redundancy reduced at the V1 layer (last layer) using different variations of the baseline model: more flexible and more rigid divisive normalization and totally rigid (totally linear) model. In both plots (left and right), error bars stand for the standard deviation over the 10 realizations of the estimation. The *theoretical estimation* also has error bars because the univariate entropies in Eq. (9) were empirically estimated

In this experiment, we compare the RBIG estimates of Δ*T* with the so-called *theoretical estimate* given by Eq. (9). In this work, redundancy reduction experiments are just instruments to illustrate the reliability of the RBIG estimations of *I*, which depend on estimating *T*. Therefore, in these instrumental experiments on Δ*T*, only deterministic responses were considered to apply the estimate given by Eq. (9). The considered layers are those in Eq. (2). Redundancy reduction was computed in the baseline model (the one leading to the best correlation with human opinion in Fig. 1 and Table 1) and also with more rigid and more flexible versions of the model. As stated before, RBIG estimations subtract $T(\boldsymbol{x})$ from $T(\boldsymbol{r}^{(1)})$, whereas the theoretical estimates accumulate the reductions Δ*T* that happen at every intermediate layer. The results are displayed in Fig. 4.

The technical conclusion is that in general the RBIG estimate of Δ*T* is consistent with *theoretical estimate*. Note that the only significant exception is for the case of the model with more flexible divisive normalization. Nevertheless, even in this case, both estimates lead to the same qualitative conclusion: this too flexible model leads to less redundancy reduction than the baseline model. This general agreement suggests that total correlation estimates (the core under RBIG information transmission estimates, as seen in Eqs. (6) and (7)) can be trusted.

From the functional point of view, it is interesting to note that redundancy reduction in the nonlinear stages of the baseline model is almost negligible. For instance, see the plateau in $\boldsymbol{r}^{(2)} \rightarrow \boldsymbol{x}^{(2)}$ (Weber-like nonlinearities) or in $\boldsymbol{r}^{(3)} \rightarrow \boldsymbol{x}^{(3)}$ (divisive normalization of texture sensors). However, it seems that this kind of nonlinear processing actually prepares the data so that subsequent linear stages can do a better job in removing redundancy. Note that the identical linear parts $\boldsymbol{x}^{(1)} \rightarrow \boldsymbol{r}^{(2)}$ (opponent transform) and $\boldsymbol{x}^{(2)} \rightarrow \boldsymbol{r}^{(3)}$ (Filters+CSFs) attain bigger reductions after the application of the von Kries nonlinearity and the Weber-like nonlinearity. This is clearer by looking at the cumulative effect at the last layer: the baseline model (in black) is substantially better than a totally linear model (in blue).

### Global experiment 2: information available at different layers of different models

This experiment compares the different image representations along the network in terms of the amount of information they share with an ideal input (noise-free LMS image). This global experiment for *I* includes the baseline model and six interesting variations: the more flexible and less flexible versions due to different divisive normalization semisaturation, the corresponding versions neglecting chromatic adaptation, and a totally linear version, which of course has no adaptation at all.

Measures at different layers include $I(\boldsymbol{r}^{(1)},\boldsymbol{r}^{(1)}), I(\boldsymbol{r}^{(1)},\boldsymbol{x}^{(1)}), \ldots , I(\boldsymbol{r}^{(1)},\boldsymbol{x}^{(3)})$. As stated in Sect. 4.2, we assume that in different representations, noise is added after a *single-step transform*, and the relative amount of noise in all the representations is the same (the deviation of the noise in each dimension is 5% of the dynamic range of that individual response). For instance, in the first case, referred to as $I(\boldsymbol{r}^{(1)},\boldsymbol{r}^{(1)})$, we compute the shared information between noise-free LMS images and LMS images corrupted with Gaussian noise with the selected SNR in each pixel and color channel.

**Figure 5:**
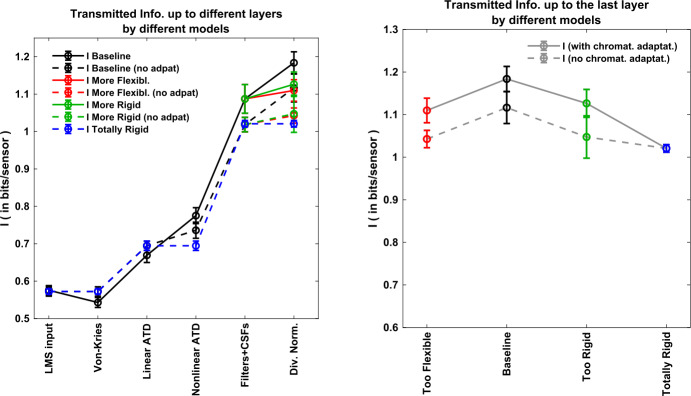
Information available from different layers and different models. Estimations assume *single-step transforms* and the same signal-to-noise ratio at every marginal sensor. *Left*: Mutual information between noise-free LMS and the noisy response at the considered layer (labels at the horizontal axis) for different variations of the model (different color/style). *Right*: Information transmitted up to the cortical layer by different variations of the model: baseline model (in black), more flexible and more rigid divisive normalization (in red and green, respectively), and totally rigid linear model (in blue). The corresponding versions of these models that neglect chromatic adaptation are represented in the same color but connected by a dashed line

Results in Fig. 5, left, show that the cortical representation (adaptive contrast in local frequency in ATD) is much better than the original representation in the spatial domain and in the LMS color space. The cortical representation doubles the amount of information captured by retinal sensors (assuming equivalent SNR). It may seem that the von Kries adaptation is not worth it (if it was the last signal representation): see how it shares less information with the input than the LMS representation for the same noise level. However, when combined with other processing blocks, the resulting representations capture more information form the input than if it was not present. These results indicate the overall progressive improvement of the stimulus representation along the pathway.

Recall that according to the noise assumptions discussed in Sect. 4.2, each point along one of the curves in Fig. 5, left, actually mean the amount of information available for a system that would perform the considered processing from the input in a single step, that is, with all the noise added at that specific layer. Therefore increasing *I* does not violate the *data processing inequality*. It just points out that these inner representations are intrinsically better because they capture more information with sensors of the same SNR quality.

These results also show that the baseline model (which gets the best correlation with human opinion in Table 1) is also the one that captures more information about the input. Note that the different modifications of the model, that is, doing it more rigid (by canceling chromatic adaptation, by doing divisive normalization more rigid, or by considering a totally linear version) or more flexible (by increasing the adaptivity of divisive normalization) leads to poorer representations in terms of transmitted information. This conclusion is clearer by looking at different versions of the last representation (after the divisive normalization) at Fig. 5, right.

The nonlinear operations both in the saturation of ATD chromatic channels and in contrast adaptation lead to significant improvements in the amount of captured information. Note that about 28% of the improvement in available information at the final representation comes from the nonlinear operations, whereas 72% of the improvement comes from the linear operations. These increments in *I* in Fig. 5, left, are interesting taking into account that these nonlinear operations make almost negligible contributions to redundancy reduction (see $\Delta T \approx 0$ for these layers in Fig. 4, left).

It is also interesting to note that spatial transforms (texture filters and contrast adaptation) definitely have a bigger contribution to the amount of captured information than chromatic transforms (von Kries adaptation, opponent channels, and Weber-like nonlinearities). Note that Fig. 5, left, shows that 67% of the improvement in available information at the final representation comes from the spatial operations, as opposed to the 33%, which comes from the chromatic operations. This is remarkable given the fact that tiny patches of 0.05 degrees were considered in our computations.

### Local experiment 1: redundancy reduction at V1 for different kinds of stimuli

In all local experiments (which involve computations over the different bins of the image space), we considered only the baseline model.

In this first local experiment, RBIG estimations of $\Delta T(\boldsymbol{r}^{(1)},\boldsymbol{x}^{(3)})$ at different locations of the image space are compared to the corresponding *theoretical estimates*. This experiment is interesting to check that the global accuracy of the information estimates illustrated in Fig. 4 actually hold for every location across the stimulus space. As stated before, experiments involving comparison with the theoretical estimate (Eq. (9)), require the use of deterministic responses.

**Figure 6:**
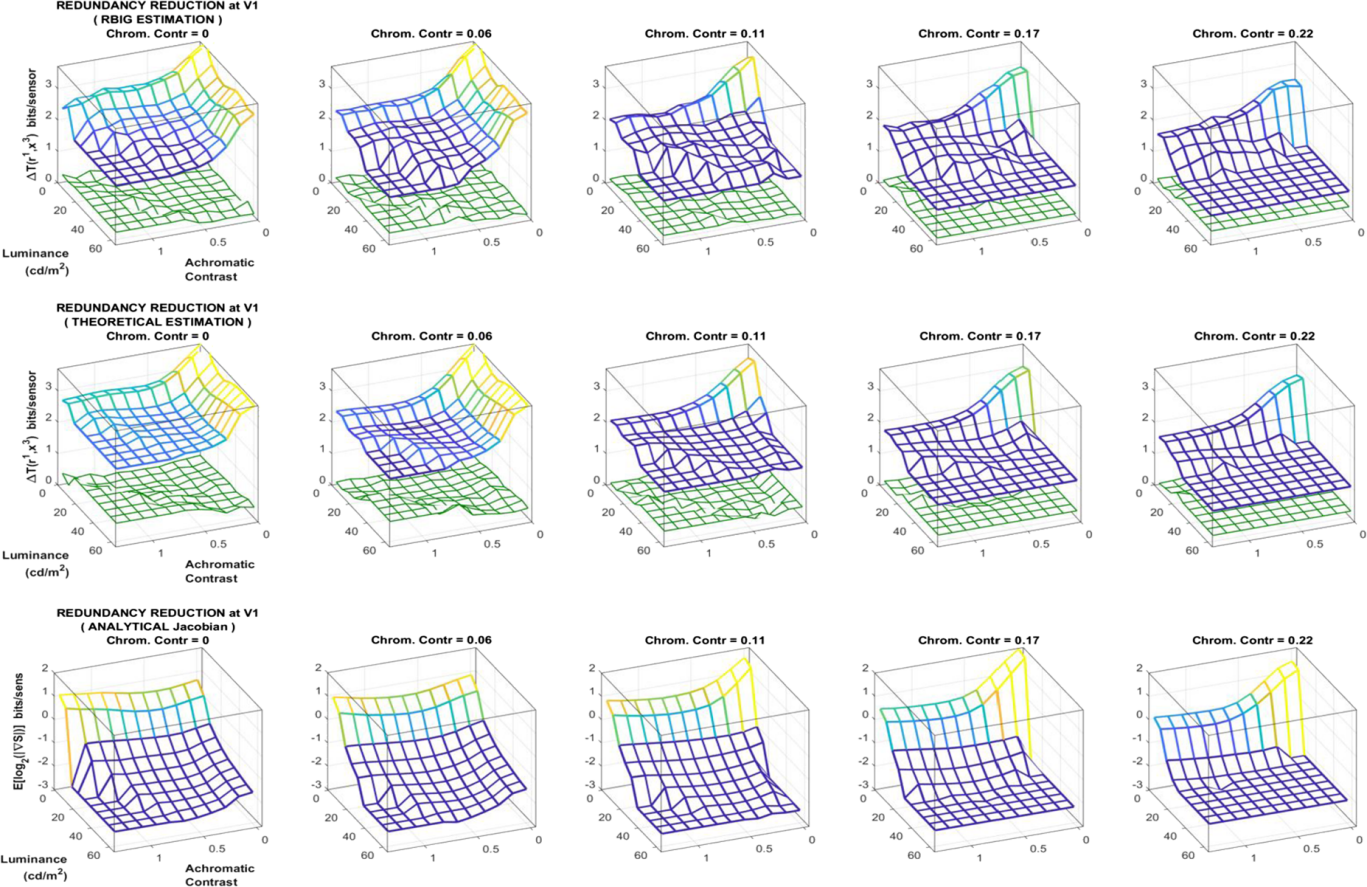
Redundancy reduction (Δ*T*, *in bits/sensor*) between the LMS input and the spatio-chromatic representation in V1, estimated via RBIG (*top row*) and via the theoretical approach of Eq. (9) (*middle row*). Results are shown for every region of the achromatic contrast/luminance space for different chromatic contrast (in different columns). The surfaces in the blue-yellow colormap are the average of 10 total correlation estimations in each case. The green surfaces at the plots of the top row represent the combination of the uncertainties of the estimates $(\sigma _{\mathrm{RBIG}}^{2} + \sigma _{\mathrm{theor}}^{2})^{1/2}$. The green surfaces at the plots of the middle row represent the absolute difference between the theoretical and the RBIG estimates. The differences are similar to the uncertainty. This agreement stresses the accuracy of the RBIG estimates and the correctness of the theoretical estimates from the analytical model. The *bottom row* shows the component of the theoretical Δ*T* that comes from the analytical Jacobian

The results in Fig. 6 show that the empirical RBIG estimate (top row) closely follows the theoretical estimate (middle row) all over the stimulus space. Note also that the difference between the estimates is small (green surfaces in the plots of the theoretical result), and this difference is similar to the uncertainties both estimates combined (green surfaces in the plots of the RBIG result). The average values of the relative difference and relative standard deviation are 0.12 and 0.09, respectively. Similar agreement is obtained for all the previous layers. Agreement of both estimates not only points out the accuracy of the proposed estimator but also the correctness of the analytical Jacobian involved in the theoretical estimate since the Jacobian of the response at the last layer includes all the previous layers.

This confirms that information estimates based on RBIG computation of total correlation can be trusted for every location of the stimulus space (not only globally as pointed out by the agreement of the estimates in Fig. 4).

Finally, the term of Eq. (9) that depends on the analytical Jacobian is shown in the bottom row. The analytical Jacobian not always represents the trends of Δ*T*. This illustrates that analytical description of the model is not enough to get complete insight on the information-theoretic performance of the model. In the specific example in Fig. 6 the analytical term roughly determines the behavior for high chromatic contrast, but it is not the case for low chromatic contrasts. This limitation of the knowledge that can be extracted from the analytical expression of the model emphasizes the need of empirical estimators such as RBIG. Section 6 elaborates on this point.

### Local experiment 2: redundancy reduction at different layers and different locations of the image space

This experiment extends the redundancy reduction values at the different layers presented in Fig. 4 to specific regions of the image space. Note that specific operations may reduce more redundancy for some stimuli than for others.

**Figure 7:**
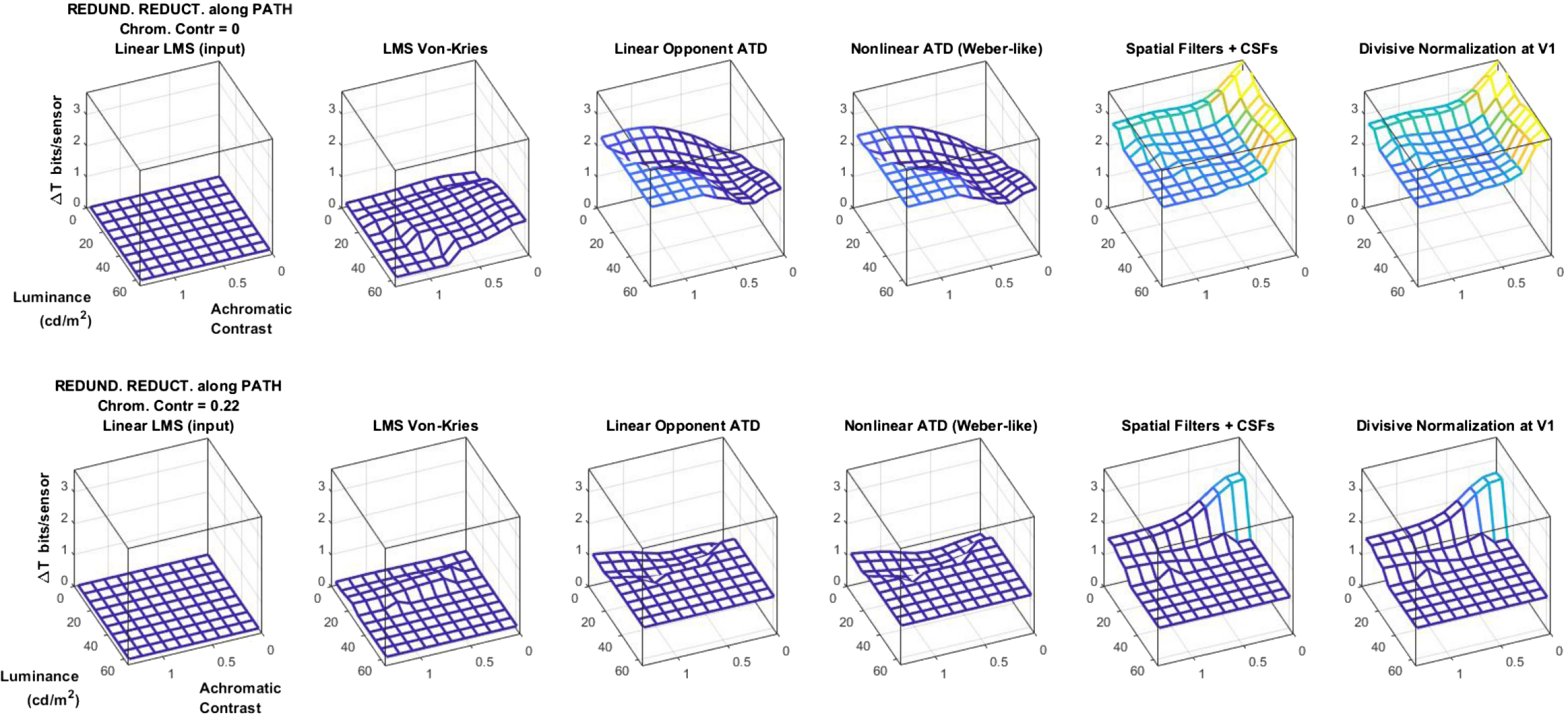
Redundancy reduction along the layers of the visual pathway (in bits/sensor). Results are shown over the achromatic contrast and luminance space for two fixed chromatic contrasts: the minimum (zero, on the top) and the maximum in out set (on the bottom). Results show that deeper image representations remove bigger amounts of redundancy

Figure 7 shows Δ*T* along the network in the achromatic contrast/luminance plane for two chromatic contrasts (the minimum and maximum in our images). We can identify general trends, which also apply to the other values of chromatic contrast not shown in the figure: (1) Δ*T* is always positive, that is, redundancy is effectively reduced by the system; (2) Δ*T* always increases along the pathway, which suggests that inner representations are better than earlier representations in terms of the information captured; and finally, (3) the increments along the way mainly occur at the linear stages, that is, the transform to opponent color representation and the transform from the spatial domain to the local-frequency domain. This relevance of linear stages in redundancy reduction is consistent to that found in Fig. 4. Note that these two linear stages are those that rotate the representation similarly to PCA (see Appendix B).

### Local experiment 3: information available at different layers and different stimuli

**Figure 8:**
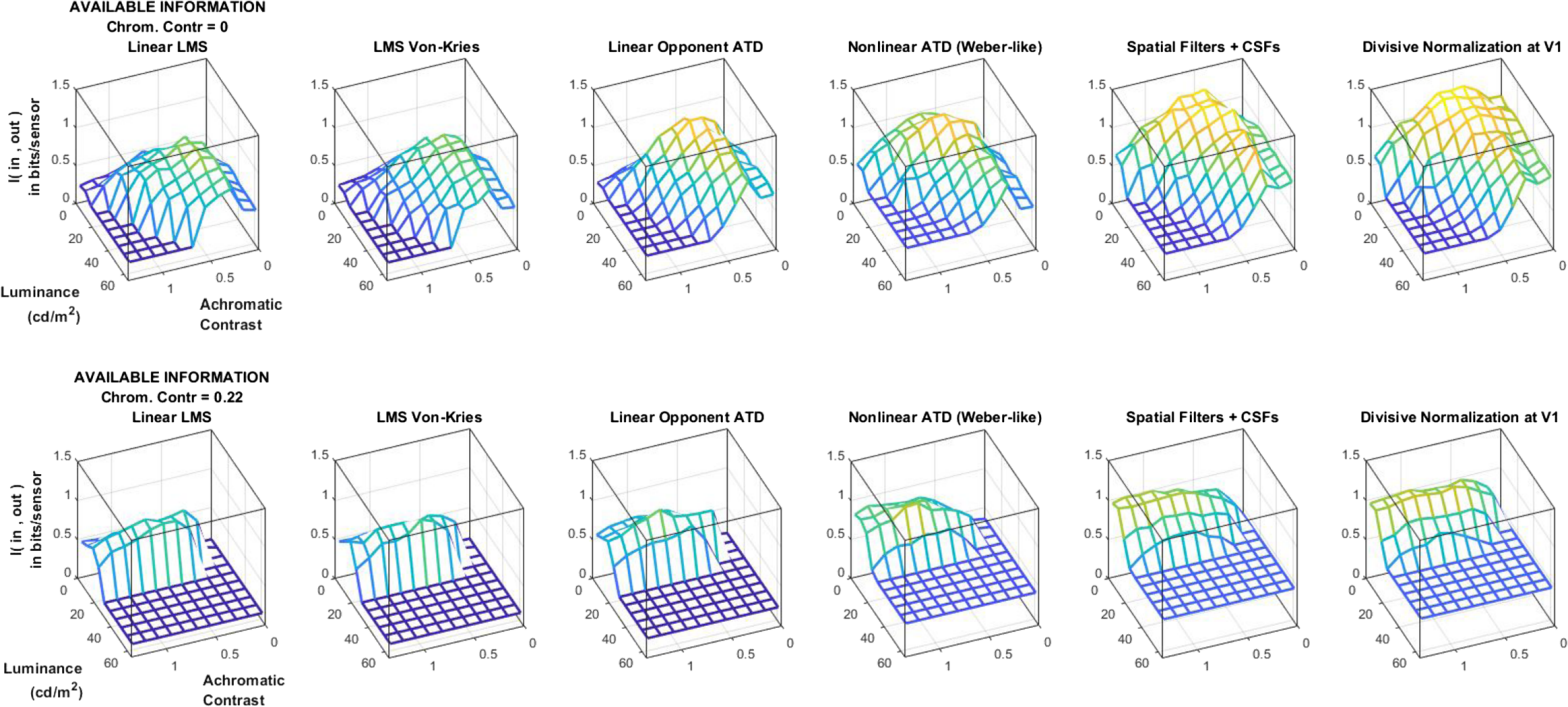
Information about the scene available from different layers of the visual pathway. Results are shown over the achromatic contrast and luminance space for two fixed chromatic contrasts: the minimum (zero, on the top) and the maximum in our set (on the bottom). In each case, we assume that sensors at the different representations have equivalent noise level (5% of the signal deviation). This result implies that using sensors of equivalent quality, the cortical representation is more appropriate because it captures more information from the input

The object of this experiment (transmitted information per unit of volume, or *per bin*, of the stimulus space) highlights the kind of stimuli better represented by different layers. Figure 8 shows the information about the scenes (in bits/sensor) available from the different neural layers assuming that the same signal-to-noise ratio of the sensors.

Only two chromatic contrasts are shown in Fig. 8, but the following trends also hold of the other chromatic contrasts omitted in the figure: assuming *single-stage* transforms and sensors with the same SNR, (1) the cortical representation is substantially better than the retinal representation since it captures more information about the scene, (2) different intermediate representations are progressively better from retina to cortex, and (3) improvements of the representation come from both linear and nonlinear stages.

Note that the total available information at certain layer (the *global* result in Fig. 5) is not the integral of the corresponding surface of *local* values in Fig. 8. However, progressive increase of the value for a region of the image space along the network can also be interpreted as an improvement of the representation. Similarly, the increments in *I* do not violate the data processing inequality given the *single-stage transform* assumption we are doing here.

It is important to note the differences of this *I* result, Fig. 8, with regard to the Δ*T* result, Fig. 7. First, whereas no substantial gain is obtained through the nonlinear stages in terms of redundancy reduction, the improvements in transmitted information due to the nonlinearities are significant. This is consistent with what was found in Figs. 4 and 5. Second, the distribution of *I* and Δ*T* over the stimulus space is substantially different; take, for instance, the bottom right plots in Figs. 7 and 8, respectively. The distribution of transmitted information seems more consistent with the statistics of the natural scenes in Fig. 3. That is the object of the comparison in the next section.

### Local experiment 4: transmitted information and the PDF of natural images

**Figure 9:**
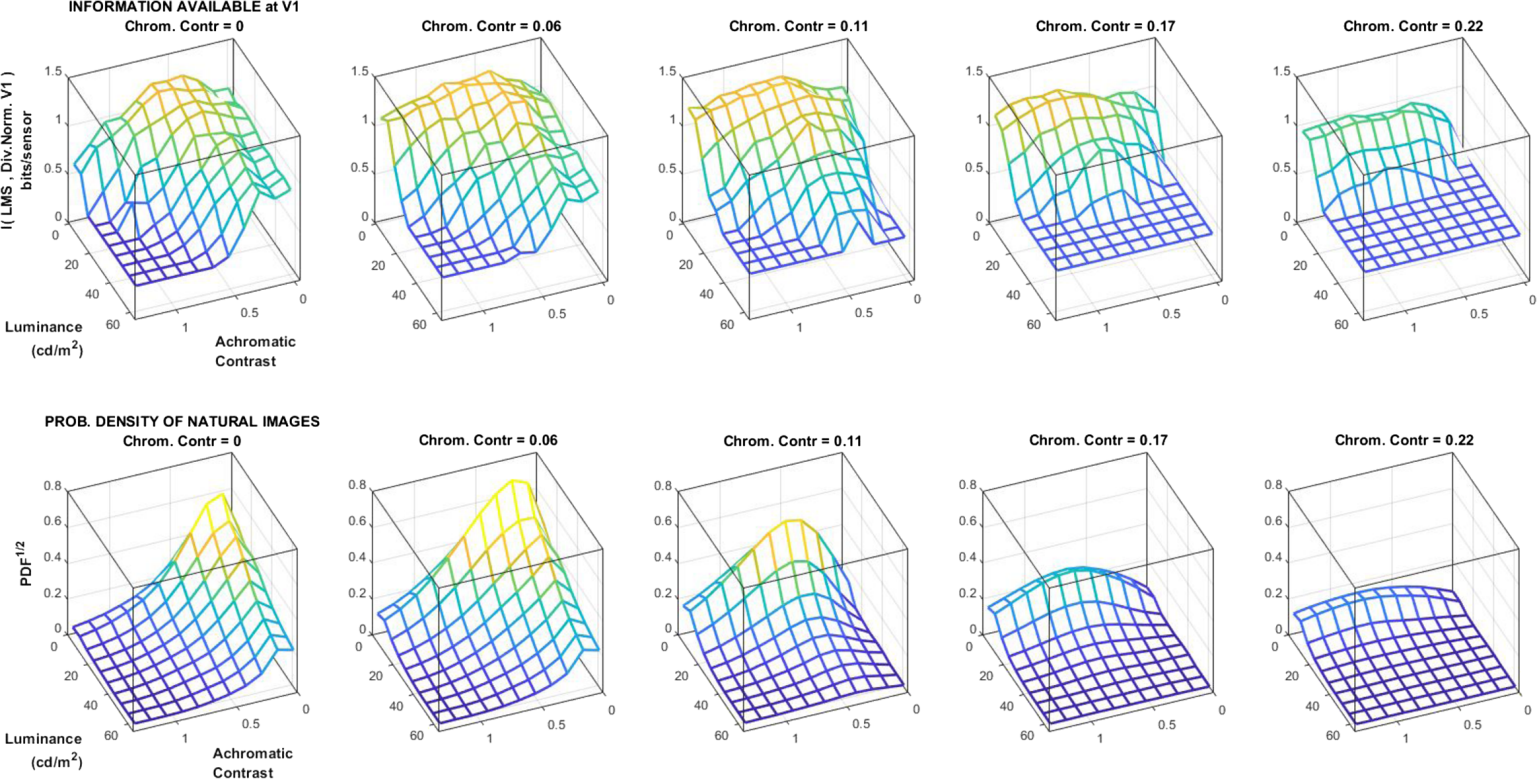
Information available at the cortical representation after divisive normalization at different regions of the image space (top) compared to the PDF of natural images (bottom)

Figure 9 explicitly compares the transmitted information up to the cortical layer $\boldsymbol{x}^{(3)}$ for stimuli at all the locations of the considered image space (top) with the distribution of natural scenes over that space (bottom).

This comparison addresses the following questions: *Does the system transmit the same amount of information for different images? if not, is this uneven transmission similar to the PDF of natural scenes?*

This result shows that the considered psychophysically tuned network (no statistical knowledge was used in this crude biological model) transmits more information in the more frequent regions of the image space: note that the peak of transmitted information shifts to higher achromatic contrasts for bigger chromatic contrasts (as the PDF of natural images), and the amount of transmitted information decreases for high chromatic contrasts (as the PDF).

This result indicates a remarkable match between the properties of the cortical representation and the relative frequency of the stimuli in the image space. Note that the amount of available information for different images is not a byproduct of the relative probability of the images. For instance, if the luminance or contrast nonlinearities were expansive (as opposed to compressive as they are), then the noise would have more impact in the low-luminance/low-contrast end. This would reduce the amount of available information for those signals using the same database to compute the results.

## Discussion and final remarks

### Efficiency of the network and hierarchy of transforms

The proposed method to estimate transmitted information allows a quantitative analysis of the different perceptual computations along the considered retina–cortex pathway.

The measurements presented above imply that the considered cortical representation captures substantially more information about the scene (factor ×2) than the retinal representation with sensors of the same signal-to-noise quality.

Not all the perceptual computations contribute to the improvement of the signal representation in the same way. Transforms acting on spatial content of the signal lead to bigger improvements in information transmission than the purely chromatic transforms carried out in the first layers (67% versus 33%). The biggest contribution is due to the analysis of ATD images through local-oriented filters and divisive normalization. The linear operations are responsible for most of the improvement in information transmission (about 72%), but the nonlinear/adaptive behavior of the system is responsible of a substantial 28%.

It is interesting that if model adaptivity is modified so that it has lower correlation with human opinion, then the amount of transmitted information reduces too. Moreover, local analysis on specific stimuli shows that the cortical representation captures relatively more information in the regions of the image space where natural images are more frequent. These two facts indicate that this biologically plausible model (not explicitly optimized for transmission) is efficient in terms of this information measure and it is well matched to the stimuli it faces.

This is in line with the approaches to the efficient coding hypothesis that go from *biology-to-statistics* [43, 44], with the advantage of using a more appropriate performance measure ($I( \boldsymbol{r},\boldsymbol{x})$ as opposed to $\Delta T(\boldsymbol{r},\boldsymbol{x})$). More generally, the proposed method can be used to estimate transmitted information in improved multigoal cost functions where constraints of different nature (energy, wiring, reconstruction error, adaptation, etc. [8–12, 26, 27, 29, 32, 36, 45]) can be taken into account. These improved multigoal cost functions can lead to interesting results in the conventional approach from *statistics-to-biology*.

### Transmitted information and analytical Jacobian

Here we discuss up to which point the trends of the transmitted information over the image space can be predicted from the analytical response. We will see that whereas the analytical Jacobian may be enough to understand the behavior of the system at a single layer (as done in [40, 43]), the relative relevance of different layers cannot be easily inferred from the corresponding derivatives. Therefore, a model-free tool such as that presented is extremely useful in this context.

First we recall the relation between *transmitted information* ($I( \boldsymbol{r},\boldsymbol{x})$, or information about the input captured by the inner image representation) and *total correlation* within the response ($T(\boldsymbol{x})$, or information shared by the responses of different sensors $x_{i}$). Whereas maximizing $I(\boldsymbol{r},\boldsymbol{x})$ is a sensible goal for a decoder devoted to solve visual problems from the response ***x***, reduction of the redundancy $T(\boldsymbol{x})$ is an alternative goal related to the maximization of transmitted information. Reasoning with $T(\boldsymbol{x})$ may be useful in some situations because it is related to the analytic expression of the perception model.

From the definitions of mutual information in terms of *entropy* and *conditional entropy* [72] (Chap. 2), $I(\boldsymbol{r},\boldsymbol{x}) = h(\boldsymbol{r}) + h( \boldsymbol{x}) - h(\boldsymbol{r},\boldsymbol{x}) = h( \boldsymbol{x}) - h(\boldsymbol{x}|\boldsymbol{r})$; and from the concept of *total correlation* [76, 77], $T(\boldsymbol{x}) = \sum_{i} h(x_{i}) - h(\boldsymbol{x})$, it is easy to see that the following expression holds:^1^

8 $$ I(\boldsymbol{r},\boldsymbol{x}) = \sum_{i} h(x_{i}) - T( \boldsymbol{x}) - h(\boldsymbol{n}). $$

Assuming that the intrinsic noise of the biological sensors cannot be reduced (and hence $h(\boldsymbol{n})$ is fixed), Eq. (8) points out *what* the system should do to increase the transmitted information: (i) it should reduce the redundancy (the total correlation *T*) within the response array, and (ii) it should increase the sum of entropies of the responses of the individual sensors. On the one hand, in the particular case of independent noise in every sensor the transform should make the responses independent to obtain $T(\boldsymbol{x})=0$. On the other hand, the entropies $h(x_{i})$ cannot be arbitrarily increased via trivial response amplification because of energy constraints. Therefore, once redundancy has been decreased as much as possible (ideally, set to zero), as the energy (or variance) of each coefficient has to be limited, the first term can be maximized by marginal equalization. Note that marginal operations do not modify the total correlation [42]. Therefore, marginal equalization could be used to maximize entropy with constrained variance (e.g., obtaining uniform or Gaussian PDFs [72, Chap. 12]) without increasing *T* and thus maximizing *I*.

Equation (8) identifies univariate and multivariate strategies for information maximization. When trying to assess the performance of a sensory system, reduction of the multivariate total correlation $T(\boldsymbol{x})$ seems the relevant term to look at because univariate entropy maximization can always be performed after joint PDF factorization through a set of (easy-to-do) univariate equalizations.

Then the *reduction in redundancy* $\Delta T(\boldsymbol{r},\boldsymbol{x}) = T(\boldsymbol{r}) - T( \boldsymbol{x})$ is a measure of performance that could be aligned with $I(\boldsymbol{r},\boldsymbol{x})$. It is interesting that for deterministic responses, this performance measure Δ*T* can be written in terms of univariate quantities and the response model [42]:

9 $$\begin{aligned} \Delta T(\boldsymbol{r},\boldsymbol{x}) = & \sum_{i} h(r_{i}) - \sum_{i} h(x_{i}) + E_{\boldsymbol{r}} \bigl\{ \log _{2} \vert \nabla _{ \boldsymbol{r}}S \vert \bigr\} \\ = & \Delta h_{m}(\boldsymbol{r},\boldsymbol{x}) + E_{ \boldsymbol{r}} \bigl\{ \log _{2} \vert \nabla _{\boldsymbol{r}}S \vert \bigr\} . \end{aligned}$$

Equation (9) is good for our purposes for two reasons: (1) *in case the marginal difference* $\Delta h_{m}$ *is approximately constant over the space of interest*, the performance is totally driven by the Jacobian of the response, so it can be theoretically studied from the model, and (2) even if $\Delta h_{m}$ is not constant, the expression is still useful to get reliable estimates of Δ*T* because the multivariate contribution may be obtained analytically from the Jacobian of the model and the rest reduces to a set of univariate entropy estimations (for which reliable estimates do exist [80]). In Sect. 5, estimates of Δ*T* using Eq. (9) were referred to as *theoretical estimation* (as opposed to model-agnostic empirical estimates purely based on Gaussianization of samples) because of this second reason. Note that as the Jacobian of the composition is the product of Jacobians, Eq. (9) implies that $\Delta T(\boldsymbol{r}^{(1)},\boldsymbol{x}^{(j)}) = \Delta T( \boldsymbol{r}^{(1)},\boldsymbol{x}^{(i)}) + \Delta T( \boldsymbol{x}^{(i)},\boldsymbol{x}^{(j)})$.

In previous works, Eq. (9) has been used to describe the communication performance either in linear systems [89] or in nonlinear transforms such as divisive normalization [40, 42] and Wilson–Cowan interaction [43]. In those cases, interesting insight could be gained from the analytical expressions of the corresponding Jacobian. In the linear case the Jacobian is constant over the image space, and therefore it is relevant when considering different linear models, but it is irrelevant when comparing the transmitted information for different stimuli. In the nonlinear cases, authors used Eq. (9) to analyze the performance at a single layer, and $\Delta h_{m}$ was explicitly shown to be roughly constant over the stimulus domain. Therefore the analytical Jacobian certainly explained the behavior of the system.

*However*, $\Delta h_{m}$ may not be constant in general, and hence the trends obtained from the Jacobian of the model can be counteracted by the variation of $\Delta h_{m}$.

In particular, the situation gets complicated if we want to study the relative effect of different layers in the cascade. At a single layer (whose Jacobian accumulates the effect of all previous layers [40]) the marginal difference of entropies *with regard to the input* may be constant over the domain and hence negligible. However, reasoning only with the Jacobians in the case of comparisons between multiple layers will only be valid if *all* the marginal differences of entropy between every layer are constant over the domain. This more strict condition is harder to fulfil in a specific network. For instance, in the illustrative model considered in our experiments, this condition not always holds (see the differences between the Jacobian and Δ*T* in Fig. 6).

Therefore, since the intuition from the analytical response is conclusive only in restricted situations, there is a need for empirical methods to estimate the transmitted information directly from sets of stimuli and the responses they elicit.

### Relation to previous work

The analysis done here has a number of relevant differences with previous work that already analyzed the statistical performance of psychophysically plausible transforms.

For instance, previous works were limited either because explored a limited range of models or because they used limited performance measures. In [89] the authors addressed the interesting study of the gain that can be obtained from different redundancy reduction transforms, basically using Eq. (9), but restricted the analysis to linear cases to neglect the term that depends on the Jacobian. In [44] the authors do consider a more general (nonlinear) model, but their analysis is limited because they did not use a multivariate measure for the redundancy, but rather a set of mutual information measures between pairs of coefficients at the considered layer.

From the technical point of view, our analysis is related to the work of Foster et al., who are also concerned about the use of accurate information-theoretic measures to study human vision [90]. The similarity is that they also use nonparametric measures of mutual information that operate directly on natural samples. The main difference is their focus on color vision and specifically on characterizing the performance of humans in different illumination conditions (e.g. determining the number of discriminable colors) [91–93]. This is related to the amount of color information in a scene that can be extracted from color measurements under other illumination [81, 90]. These problems are related to entropy and mutual-information measures (which is the same problem that we address with RBIG), but they do not quantify the information flow through the visual pathway (mutual information between layers and redundancy within layers) that we address here to identify the most relevant computations. As an example, in [92, 94] the redundancy is considered only because of its impact on the available information for illumination compensation, but not as a measure of information transmission in the visual pathway.

An example of the conceptual difference is that chromatic transforms actually are less important for information transmission than spatial transforms when considering spatio-chromatic aspects at the same time. Moreover, note that the von Kries color adaptation transform is actually the only transform leading to a representation that captures less information than the input representation (for sensors with the same SNR). Color adaptation may be more related to manifold alignment to improve color-based classification (the kind of goal studied by Foster et al.) than to improve information transmission (the goal we study here). Despite these differences, the interesting improvements of Kozachenko–Leonenko entropy estimator [79] proposed in [81] should be compared in the future with RBIG because these two alternative estimators may be applicable to other problems of visual neuroscience [95].

This work originated from the analytical results for total correlation developed for cascades of linear+nonlinear networks [40] and from the analysis of redundancy reduction in Wilson–Cowan networks [43]. In both cases the analysis was restricted to achromatic stimuli. In [40] the approach was totally analytical, whereas [43] included RBIG estimations for the first time. However, the main difference is that those works considered not the transmitted information, but the redundancy reduction surrogate. In this work, we show that, in general, Δ*T* may not be a good descriptor for *I*.

### Consistency between different databases and models

Although [43] is purely achromatic and does not consider *I* (which are crucial conceptual differences), comparison with those results is interesting for different reasons: (1) results of the redundancy at the input retinal representation are comparable (beyond the achromatic/chromatic difference), and some interesting consequences can be extracted; (2) the small gain in redundancy reduction at the Weber saturation and at the divisive normalization saturation (also obtained in [43]) has been better explained here.

First, it is interesting to note that this work and [43] use different databases: the colorimetrically calibrated IPL database [27, 32] and the radiometrically calibrated database by Foster, Nascimento, and Amano [69, 70] respectively. It is interesting that (for the users of the databases) the redundancy measures at the retinal input are comparable, which means that the statistics of both databases is similar. Specifically, for achromatic patches subtending 0.06 deg in the Foster et al. database the total correlation is about 3.8 bits/sensor [43]. Here, for color patches subtending a smaller angle (0.05 deg in the IPL database), the total correlation is 4.1 bits/sensor. This redundancy is a little bit bigger because it includes color, which is redundant, but not that big because the size is smaller, and hence less spatial structure is present, which should increase redundancy too. Moreover, this suggests that consideration of color on top of spatial information increases the redundancy by a small amount, which is consistent with the fact that spatial operations are more relevant in removing redundancy and transmitting information.

Second, the models considered in both works have similar structure, but they are not exactly the same: the one here has no specific layer for contrast computation and preserves the dimension in the local frequency transform. However, in both cases, small gain in Δ*T* is obtained at the Weber-like saturation and at the cortical divisive normalization. This consistency of behavior is a safety check for the models, but it is more interesting that the analysis of *I* proposed here and the point made about the advantage of using *I* as descriptor of performance explains the benefits of these saturations even though they do not contribute to the reduction of redundancy.

### Further work: consequences in image quality metrics and image coding

The *visual information fidelity* (VIF) [96, 97] is an original approach to characterize the distortion introduced in an image, which is based in comparing the information about the scene that *a human* could extract from the distorted image with respect to the information that he/she could extract from the original image.

The results presented here can be incorporated in that attractive framework in different ways. On the one hand, we may improve the perceptual model including nonlinearities and more sophisticated noise schemes with no restriction because the nonparametric RBIG estimation is insensitive to the complexity of the model. On the other hand, estimations of mutual information in the original VIF scheme made crude approximations on the PDF of the signals to apply analytical estimations, which may be too biased. Better measures of *I*, not subject to approximated image models, do improve the original VIF results [98].

Following previous tradition of improvements of JPEG/MPEG compression based on divisive normalization [62, 99], current state-of-the-art in image coding also uses this kind of perceptually inspired linear+nonlinear architectures [100]. The difference is that current architectures are optimized through the powerful automatic differentiation tools refined for deep-learning [101]. In this case the encoding and decoding transforms are optimized to minimize simultaneously the *bitrate* and the *perceptual distortion*. Nowadays these two magnitudes have different nature. However, with the considerations done here, VIF distortion, which is expressed in information-theoretic units, could have more meaningful values, and the rate in the image coder could be bounded or modulated by the amount of information transmitted by the perceptual system, thus leading to a better optimization goal.

## Figures and Tables

**Figure 10:**
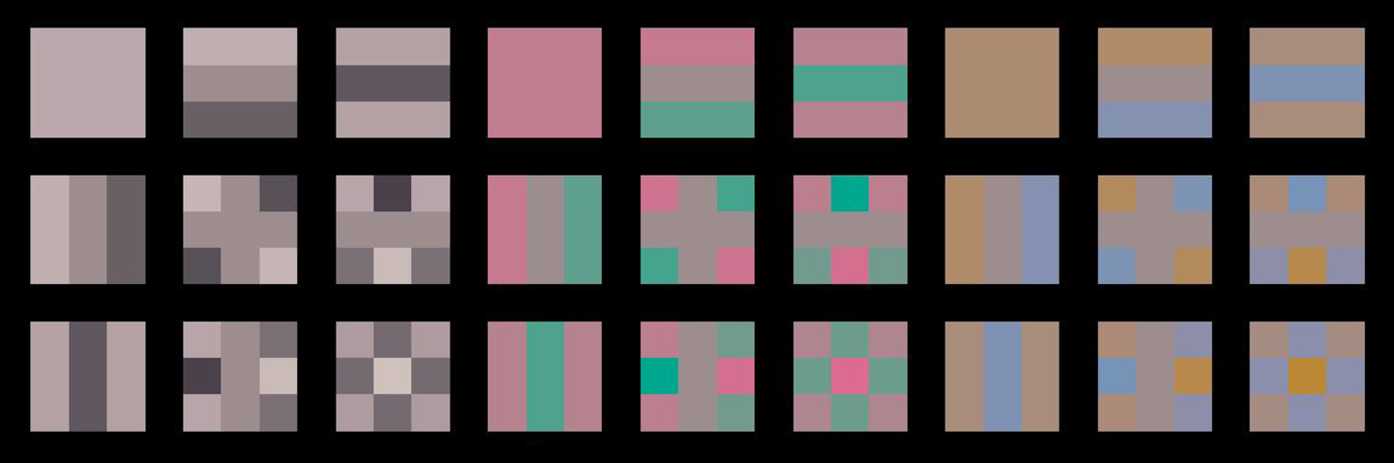
Receptive fields of the spatio-chromatic texture sensors used in our implementation

**Figure 11:**
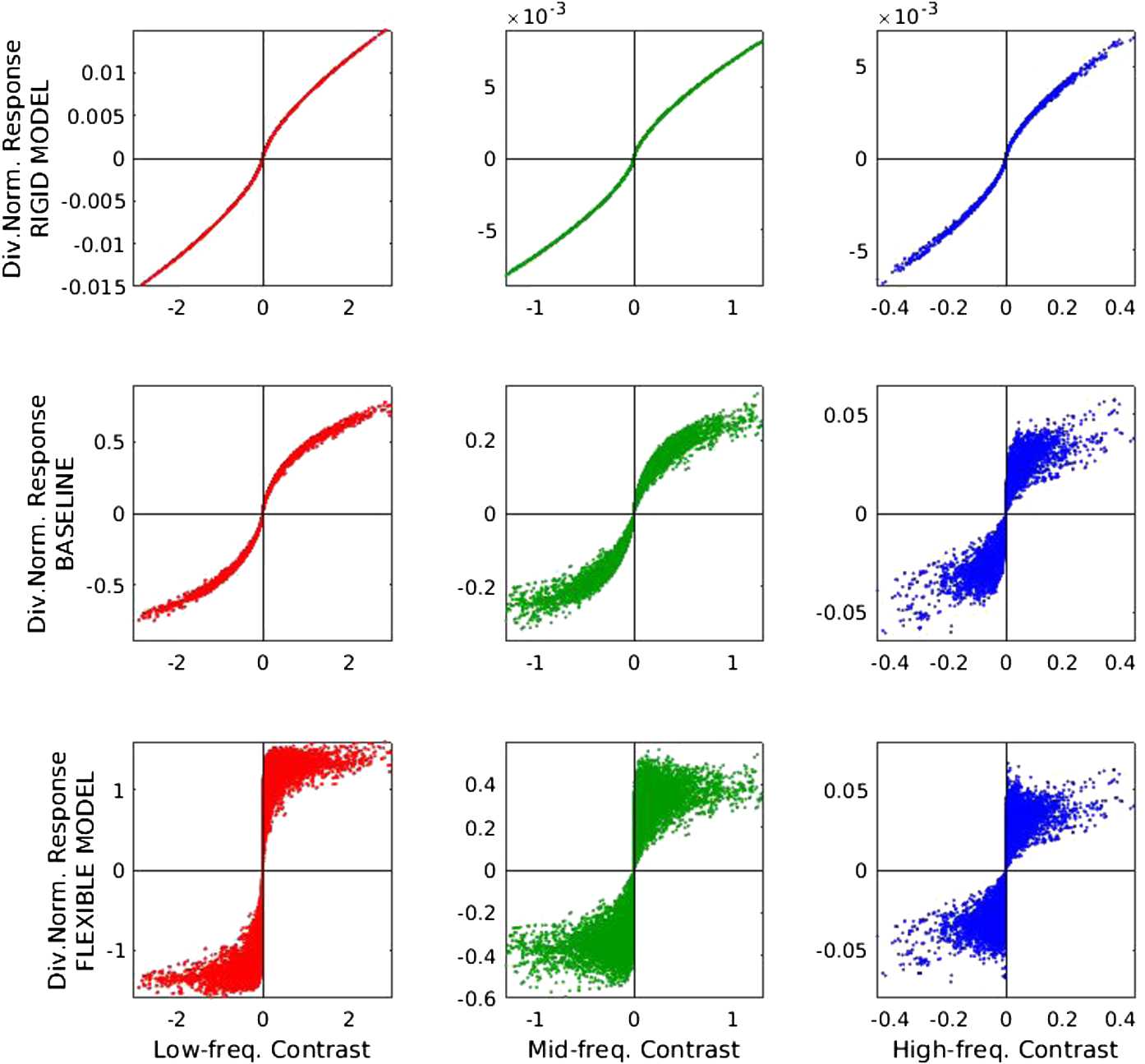
Flexibility of the nonlinearities of the texture sensors as a function of the semisaturation constants. Here flexibility is modified in different rows by changing the semisaturation *b* in the divisive normalizations ($100 \times b$ in the first row and $0.01 \times b$ in the third row). In these plots, we show the responses of texture sensors tuned to low, medium, and high frequencies (left, center, and right columns respectively) for multiple images (each point in the scatter plots is one image). The central row shows the response of the sensors in the *baseline* model. Note how the nonlinearity is image-dependent, and hence there is no a single response curve, but rather a scatter plot. As the flexibility is increased (from the top row to the bottom row), the response goes from a fixed curve to a wider family of curves

**Figure 12:**
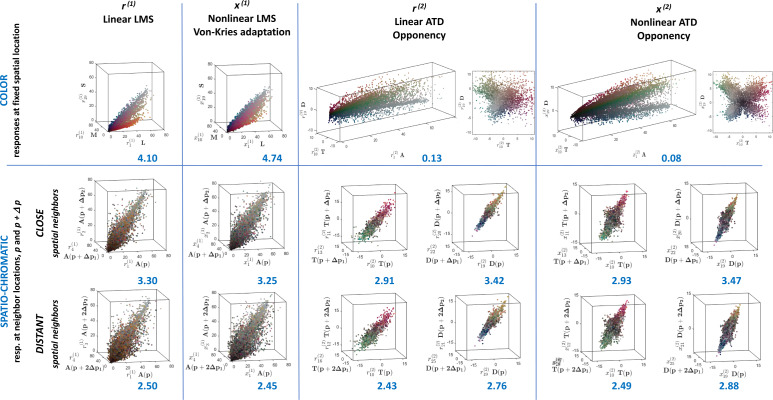
Image manifold transformed through the chromatic layers. Different rows display projections that represent different aspects of the signal, and different columns show how these aspects transform over the series of layers of the network. In the first row the samples are colored according to the actual color they represent (appropriate rendering was done with Colorlab [102]). The scatter plots in the spatio-chromatic rows actually represent 4 dimensional data: the sample in a point in the 3d-axes represents the response of the corresponding chromatic sensor at the three corners of a square, and the color of the sample corresponds to the color of the fourth corner in the square. Correspondence of the color of the sample with the location in the space implies strong correlation between spatial neighbors. Numbers in blue display the 2nd-order correlation measure proposed in [105]: $C = \frac{1}{2}log_{2}(\frac{\prod_{i} \Sigma _{ii}}{|\Sigma |})$, where Σ is the covariance matrix, and hence *C* is the difference between the entropy of the marginal distributions and the joint entropy assuming Gaussian approximations

**Figure 13:**
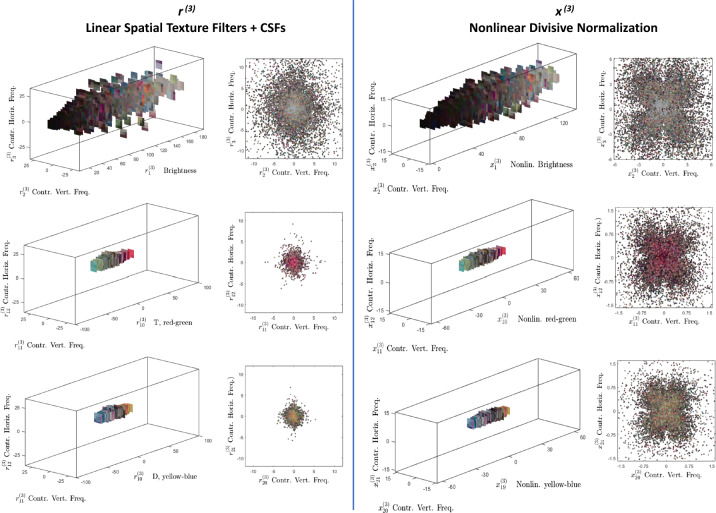
Transform of the image manifold through the spatial layers. In the 3*d* scatter plots the stimuli (image patches) are displayed at the locations given by the responses they elicit in the three considered sensors. This illustrates the texture-chromatic meaning of each region of the spaces. On the contrary, 2*d* projections only display dots of the average color of the corresponding patches so that the *elliptically symmetric* shape or the *four-leaf-clover* shape of the densities can be seen

**Figure 14:**
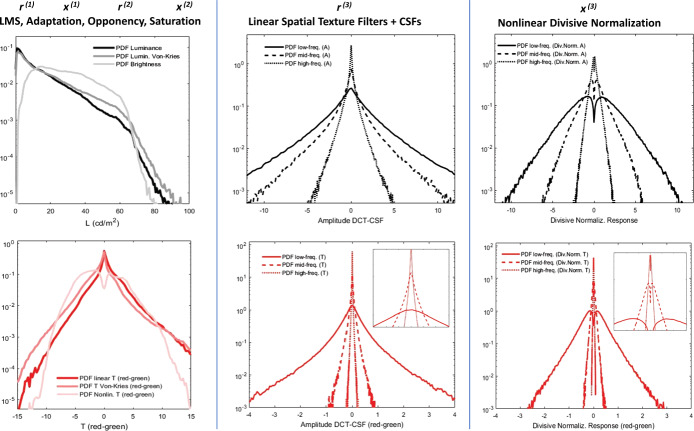
Marginal PDFs of chromatic and spatial-texture sensors along the visual pathway

**Figure 15:**
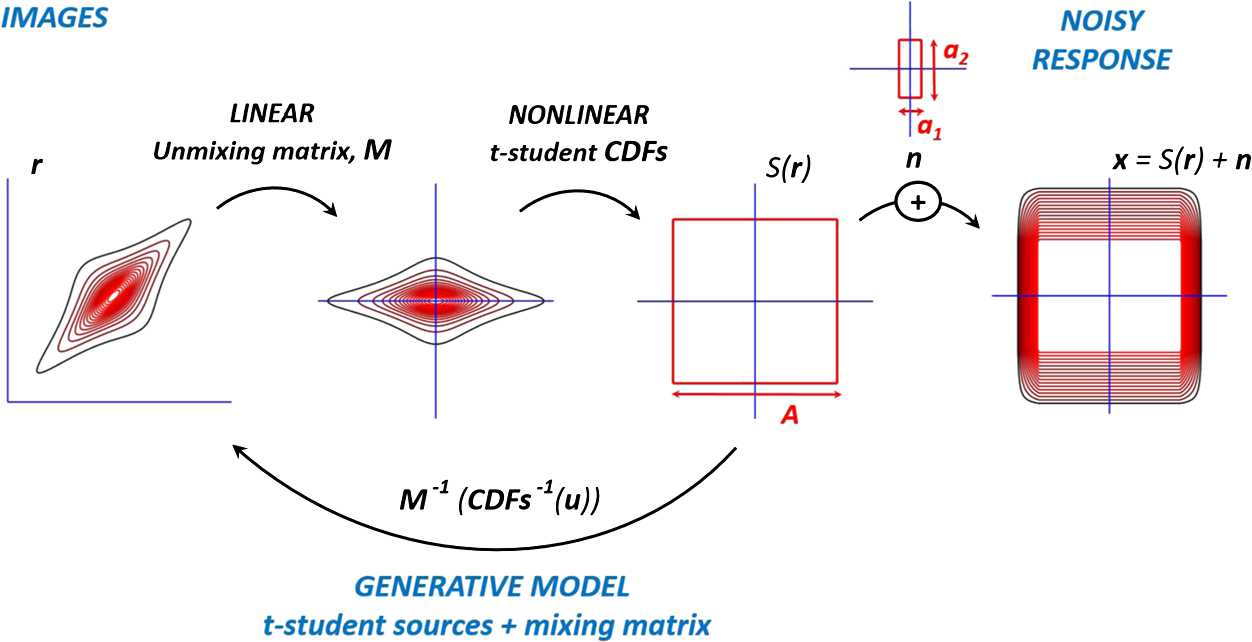
Idealized image model and sensory system. (1) A simple analytical expression for transmitted information, $I(\boldsymbol{r},\boldsymbol{x}) = \sum_{i=1}^{d} ( \frac{A-a_{i}}{A} \log _{2}(A) + \frac{a_{i}}{A} \log _{2}(e^{1/2} A) - \log _{2}(a_{i}) ) $; (2) a simple procedure to generate image samples to check the information estimators

## Data Availability

The color-calibrated image database is at http://isp.uv.es/data_calibrated.html. The code for the visual model is at https://isp.uv.es/code/visioncolor/infoDN.html. The RBIG estimator is at http://isp.uv.es/RBIG4IT.htm.

## Appendix A: Equations of the standard vision model

In this appendix, we list the equations for the *linear* and the *nonlinear* parts of the layers. The linear transforms in the model are always of the form

10 $$ \mathbf{r}^{(i)} = L^{(i)} \cdot \mathbf{x}^{(i-1)}, $$

where the matrix $L^{(i)}$ depends on the meaning of the layer. The input layer transforms the spectral radiance in each spatial location into the LMS tristimulus vectors. In this case the matrix $L^{(1)}$ is made of the color matching functions of LMS cones [46].

The linear part of the second layer transforms von Kries-adapted LMS responses into ATD channels. In this case, the matrix $L^{(2)}$ is the linear change of basis from LMS to ATD, and in our model, we chose the Jameson and Hurvich ATD space [47].

The linear part of the third layer transforms the Weber-saturated ATD images into a local-frequency representation, and the responses of different frequencies are weighted according to the CSF. Therefore $L^{(3)} = \Lambda _{CSF}\cdot B$, where the rows of the matrix *B* contain the basis functions of a local 2D discrete cosine transform, and $\Lambda _{CSF}$ is a diagonal matrix with the weights corresponding to each frequency and color channel. The shape of the receptive fields (basis functions or texture sensors) in *B* is shown in Fig. 10. Here the sensitivities for the achromatic and the chromatic channels in the Fourier domain [56, 57] are transformed into the cosine domain according to the procedure described in [59].

The first nonlinear transform performs von Kries adaptation [20]:

11 $$ \mathbf{x}^{(1)} = M\bigl(\mathbf{r}^{(1)}\bigr) \cdot \mathbf{r}^{(1)}, $$

where the scaling matrix is diagonal with $M(\mathbf{r}^{(1)})_{ii} = \frac{T_{i}(\textrm{canonical})}{T_{i}(\textrm{scene})}$, where $T_{i}$ are the tristimulus values of what is considered to be white in the input scene and in a canonical scene.

The second nonlinear transform is a Weber-like saturation [20, 50, 51], which here is implemented as a simple pointwise exponentiation (with $\gamma <1$):

12 $$ \mathbf{x}^{(2)} = \operatorname{sign}\bigl( \mathbf{r}^{(2)}\bigr) \cdot \bigl\vert \mathbf{r}^{(2)} \bigr\vert ^{\gamma}. $$

The third nonlinear transform is implemented using the standard divisive normalization [18]:

13 $$ \mathbf{x}^{(3)} = \operatorname{sign}\bigl( \mathbf{r}^{(3)}\bigr) \cdot K \cdot \frac{ \vert \mathbf{r}^{(3)} \vert ^{\gamma}}{b + H \cdot \vert \mathbf{r}^{(3)} \vert ^{\gamma}}, $$

where the stabilization constant *K* and the interaction kernel *H* are computed as proposed in [41] to scale the responses of each subband as in the local-frequency domain. This is related to the underlying Wilson–Cowan implementation of divisive normalization [64]. Note that the vector of semisaturation constants *b* controls the flexibility of the nonlinearity. If *b* is reduced, then the signal-dependent term in the denominator is more relevant. On the contrary, very large semisaturations imply a rigid nonlinearity. This is illustrated in Fig. 11.

Our implementation is based on the Matlab libraries Colorlab [102] and Vistalab [103], and it is available at http://isp.uv.es/code/visioncolor/infoDN.html.

## Appendix B: Manifold of images through the visual pathway

Here we present scatter plots of the spatio-chromatic samples at different layers of the considered model and the marginal PDFs throughout the network. These transforms illustrate in practice the comments made in Sect. 6 on Eq. (8): to increase the transmitted information, redundancy between the responses at each layer throughout the pathway should be reduced, and the marginal PDFs should be equalized.

Moreover, visualization of the marginal PDFs and the corresponding joint PDFs is interesting in case we want to propose models for the marginals, as in [44, 104], to make analytical estimations of the marginal entropies in Eqs. (8) or (9).

Figure 12 shows the geometrical effect of the pointwise chromatic transforms that occur at the first layers of the network. We display selected projections of the 27-dimensional arrays to illustrate (1) the distribution of the chromatic information (response of color sensors, LMS or ATD, at a fixed spatial location, in the scatter plots of the first row) and (2) the distribution of the spatio-chromatic information (response of color sensors, A, T, and D tuned to different spatial locations, either close spatial neighbors (middle row) or more distant neighbors (bottom row)).

In the input representation of LMS or $\boldsymbol{r}^{(1)}$ vectors, the responses are strongly correlated, both between sensors of different spectral sensitivity and between sensors tuned to different spatial locations (see the alignment of the scatter plots and the numbers *in blue* representing 2nd-order correlation). This represents the spectral [106, 107] and spatial [108] smoothness of natural scenes. As no chromatic adaptation has been applied yet, the scenes under reddish illumination lead to a distinctly separated blob in the color manifold (top plot of the first column). Note also the lower length of this cluster due to the lower luminance of the CIE A illumination in the experimental setting. In the spatio-chromatic rows (still the first column), note that alignment of the scatter plot corresponding to sensors that are closer in space is bigger, consistently with a bigger correlation measure. Not surprisingly, spatial smoothness decays with distance.

Divisive normalization by the *white* (von Kries adaptation, 2nd column) aligns the distinct clusters corresponding to the CIE D65 and CIE A in the input representation (see scatter plot and increased correlation measure), but it does not introduce qualitative changes in the distributions with spatial information.

Transform to opponent channels (3rd column) substantially reduces the correlation between color sensors at a fixed location. It is remarkable how Jameson and Hurvich opponent transform rotates the manifold as a sort of PCA even though it is not based on any statistical consideration. This is consistent with efficient coding interpretations of this stage [24]. Nevertheless, responses of chromatic sensors T and D are still strongly correlated to their spatial neighbors. In fact, the spatial correlation of the chromatic sensors is similar to the achromatic counterpart (in previous columns). In the case of chromatic sensors, spatial correlation also decays with distance.

Nonlinear saturation of the response at each chromatic sensor (4th column) reduces the correlation even further. Note that here we just picked a crude exponential saturation with fixed exponent following the Weber-like curves of the brightness and opponent mechanisms [50, 51]. However, this psychophysically inspired choice reduced the correlation again, consistently with efficient coding interpretations of this nonlinearity [26, 27]. However, as in the previous layers, this chromatic transform also has small effect on the spatial interaction between the sensors (mid and bottom rows of the 4th column). Finally, it is important to mention the effect of this saturation in the joint PDF: note the *four-leaf-clover* shape of the color manifold projected onto the nonlinear T-D plane (top-right scatter plot). This *four-leaf-clover* shape is a characteristic consequence of the saturation since it also appears in the spatial transforms based on divisive normalization and has consequences on the bimodal nature of the marginal PDFs (see Figs. 13–14) reported before [44].

Figure 13 illustrates the effect of the spatial transforms: texture sensors with local oriented receptive fields tuned to different frequencies weighted by achromatic and chromatic CSFs (linear responses $\boldsymbol{r}^{(3)}$) and divisive normalization (nonlinear responses $\boldsymbol{x}^{(3)}$).

In each case, linear and nonlinear, on the left and right panels, respectively, we display samples in 3*d* spaces corresponding to sensors tuned to zero frequency and to two low-frequency components of vertical and horizontal orientation. We also represent samples in 2*d* projections for a better assessment of the shape of the joint PDF. This is done for three kinds of chromatic sensors: achromatic, red-green, and yellow-blue (on the top, med, and bottom rows, respectively).

In this case, the 2nd-order correlation measure *C*, given in the previous figure (numbers in blue), was not included because here differences are more subtle: *C* is basically negligible in all cases, and differences are in the level of the estimation error. Actually, differences between these representations have to be described by more appropriate higher-order (multivariate) estimates proposed in this work, the total correlation, and the transmitted information.

The scatter plots in the first column illustrate how the texture sensors reorient the PDFs of the spatial neighbors of the previous layers. In the bottom rows of the previous figure the PDFs were systematically correlated regardless of the chromatic manipulations. The spatial transforms applied to the A, T, and D parts of the array $\boldsymbol{x}^{(2)}$ virtually remove the 2nd-order correlation. This is consistent with the efficient coding interpretation of this linear filter bank [28–32].

The top left scatter plot shows that stimuli with specific visual features actually give the expected response because it is located in the corresponding region of the space: note, for instance, the horizontal/vertical patterns of high contrast and different polarity aligned along their corresponding axis and located along the zero-frequency axis according to their brightness.

As always throughout this Appendix, equivalent plots represent the same sets of samples. Therefore the scatter plots of the second and third rows (still left column) represent the very same set of images as the top-left scatter plot. However, the location of these images in the chromatic texture axes is markedly different because of two reasons: (1) chromatic contrast is smaller than achromatic contrast in natural images (note that images with high chromatic contrast in Fig. 3 are less frequent), and on top of that; (2) the chromatic CSFs has lower bandwidth than the achromatic filter, so the high frequency color oscillations are strongly attenuated by the system. As a result, the images are clustered along the DC color axis. This difference in amplitude of the oscillations can be seen in the extent of the samples over the 2*d* scatter plots on the left panel. It is interesting that in these 2*d* plots, we can see that the elliptically symmetric distribution, reported before for natural achromatic images [42], also happens in the T and D channels.

The right panel shows the result of a divisive normalization transform applied to the responses of the linear sensors. Divisive normalization implies saturation in each dimension, but (as opposed to the crude dimensionwise saturation done in the chromatic channels) here the saturation of the response of a sensor for certain stimulus depends on the activity of the sensors tuned to other textures [22, 62]. Saturation is apparent in the 3*d* scatter plots because the higher contrast patterns in the achromatic plot have been pushed toward the DC axis and the low-brightness region has been expanded. In the chromatic cases, saturation is also apparent since the low-saturation region has been expanded.

The 2*d* scatter plots show how, due to divisive normalization, the elliptically symmetric distribution characteristic of local-frequency representations change to this *four-leaf-clover* shape, as in the case of the color distribution after the saturation (previous figure, top-right scatter plot).

Figure 14 shows that saturation operations also lead to bimodal marginal PDFs for chromatic sensors together with classical results for marginal PDFs [25, 104, 108–110]. These bimodal marginal PDFs had only been reported for achromatic texture sensors [40, 44].

Regarding well-known results, Fig. 14 (top left) shows how the first layers contribute to equalization of the PDF of the responses associated to brightness: von Kries adaptation expands the range of the responses to the stimuli under darker illumination, and the saturation contributes to reduce the peak at the dark region [25, 109]. In the chromatic case (bottom left), before von Kries adaptation the tail in the reddish region is heavier (half of the samples are under the reddish CIE A illumination). Then adaptation reduces the bias in the PDF, and saturation leads to a bimodal PDF of reduced support in the red-green sensors (yellow-blue sensors behave similarly). The column at the center displays heavy-tailed PDFs in the case of linear texture sensors, either achromatic (center top) or chromatic (center bottom), with decreasing variance depending on the frequency [104, 108, 110].

On the other hand, the right column and bottom-left plot of Fig. 14 show how the joint distributions with a mode in each quadrant (*four-leaf-clover* shapes) in Figs. 12 and 13 lead to the bimodal marginal PDFs. These marginal PDFs are not specific of the considered dataset: they appear in the van Hateren radiance calibrated dataset and in the Foster–Nascimento–Amano dataset [69, 70], as reported in [43, 44], respectively. This shape appears when applying divisive normalization saturations with appropriate exponent: in [44], we proposed a functional form for this marginal related to the parameters of the divisive normalization, and this kind of *four-leaf-clover* joint PDFs and resulting bimodal marginal also appear when optimizing divisive normalization for image coding [100] (personal communication from the authors).

According to Eq. (8). analytical modeling of these marginal PDFs can be helpful to characterize the performance. However, in this illustrative section, we just want to show new evidences (now for chromatic sensors) about the fact that the family of possible PDFs after divisive normalization is not restricted to Gaussian, as sometimes assumed [111].

The suggestions made in this Appendix are actually quantified using the proposed Gaussianization tool in Sect. 5.

## Appendix C: Performance of RBIG estimators of *I* for *t*-student and Gaussian sources

In this appendix we justify the use of a specific RBIG estimator for the transmitted information (Eq. (7)), for image-like sources ***r*** and sensory-like systems $\boldsymbol{x} = s(\boldsymbol{r}) + \boldsymbol{n}$. As a convenient reference, we also consider the transmitted information in the trivial case $\boldsymbol{x} = \boldsymbol{r} + \boldsymbol{n}$ when both signal and noise are Gaussian.

Natural images are known to be non-Gaussian since empirical image histograms are found to have heavier tails than Gaussian PDFs (see Appendix B and [104, 108, 110]). This motivated the use of different sorts of models (such as Gaussian scale mixtures [112], mixtures of heavy-tailed distributions with controlled correlation [33], $L_{p}$-symmetric densities [113], Gaussian densities with signal-dependent covariance [44], etc.). In this appendix, we assume a simpler image model based on *t*-*student* densities defined in a local frequency domain. We do this for convenience in sample generation and in the computation of an analytical ground truth for transmitted information. Image models based on *t*-*student* have been used in natural image coding [114], and they are a common illustrative example when discussing the properties of natural images [32, 42, 44]. Another advantage of *t*-*student* models is that they can be turned into Gaussian PDFs by increasing the degrees-of-freedom parameter *ν* that controls the kurtosis of the density.

Figure 15 describes both a simple linear+nonlinear noisy sensory system and a procedure for sample generation. For a simple computation of the transmitted information, the linear and nonlinear stages of the sensory system match the elements of the image model. First, the linear transform identifies the independent components of the images (because it uses the inverse of the selected mixing matrix), and then it marginally equalizes the outputs (because it uses the marginal cumulative density functions (CSFs) of the selected *t*-*student* PDFs to lead to a joint uniform PDF, a *d*-cube of size *A*). Finally, the response in this uniform domain is subject to uniform noise, eventually with different amplitudes $a_{i}$ for each coefficient. The advantage of this idealized setting is that the transmitted information can be computed analytically and samples are easy to obtain with proper choices for the transforms.

In this setting, synthetic images are obtained in the following way. First, samples drawn from the uniform density in the *d*-cube are passed through the inverse univariate CDFs of the selected *t*-*student* PDFs to obtain independent univariate heavy-tailed samples. Then these independent sources are mixed using a $d\times d$ *mixing* matrix. In order to have sensible image samples, the mixing matrix for this illustration is the inverse of three sets of 2D-DCT filters of size $3\times 3$ ($d=27$ as in the experiments done with real images). The *t*-student sources are defined in a color-opponent space (the same used in the model). Therefore the resulting opponent images after the inverse DCTs are linearly mixed again according to the ATD-to-LMS matrix to finally obtain $9\times 9$ image patches in the LMS color space. Note that, given the known frequency and color meaning of the *i*th column of the mixing matrix, if the amplitudes of the *t*-*student* variables are multiplied by a frequency and color dependent factor, then we can reproduce the classical $1/f$ spectrum of natural images [104, 109, 110, 115] and the relative energy of the opponent channels [30].

The analytical transmitted information is also easy to compute using Eq. (8). First, the joint entropy of the noise is $h(\boldsymbol{n}) = \sum_{i_{1}}^{d} \log _{2}(a_{i})$ because it is drawn from a *d*-rectangle of sizes $a_{i}$. Second, we see that $T(\boldsymbol{x})=0$ because the components of the noisy response are independent since the PDFs of $s(\boldsymbol{r})$ and ***n*** are uniform *d*-rectangles aligned with the axes. Finally, the computation of $\sum_{i=1}^{d} h(x_{i})$ is also easy because noise additivity implies that the marginal PDFs are the convolutions of the PDFs of the deterministic response and the noise. That means the convolution of two unit-volume rectangles of support *A* for the response and $a_{i}$ for the noise. This leads to marginal PDFs with truncated-pyramid shape. Therefore the entropy integral can be broken into the known entropy integrals of a *rectangle* and a *triangle*, and hence $h(x_{i}) = \frac{A-a_{i}}{A} \log _{2}(A) + \frac{a_{i}}{A} \log _{2}(e^{1/2} A)$ for the case $a_{i}< A$. These amplitudes have to be interchanged if $a_{i}>A$.

An analytical solution is also possible in the trivial case (Gaussian source, identity transform, and Gaussian noise independent of the response). In that case, using that $I(\boldsymbol{r},\boldsymbol{x}) = h(\boldsymbol{x}) - h( \boldsymbol{n})$ and the known entropy of the Gaussian, we have $I(\boldsymbol{r},\boldsymbol{x}) = \frac{1}{2} \log _{2} ( |2 \pi e (\Sigma _{r} + \Sigma _{n})| ) - \frac{1}{2} \log _{2} ( |2 \pi e \Sigma _{n}| )$.

Table 2 shows the relative error with regard to the analytical value (in percentage of the actual information) in the two cases, *t*-*student* and *Gaussian*. We compare the RBIG-based expressions (7) and (6) with different implementations of classical mutual information estimators based on k-NN estimators of entropy [79, 80] and a recent improvement of the Kozachenko–Leonenko estimator [81]. Different values of the kurtosis (from more image-like $\nu = 3, 4$ to more Gaussian $\nu =100$) are explored, as well as different number of samples in the estimation.

**Table 2:** Performance of *I* estimators for *t*-*student* and *Gaussian* samples (Relative Error in %)

Shape	# Samples	Eq. (7)	Eq. (6)	KL+offset [81]	KL [79]	k-NN [80]
T-STUDENT source/Ideal Infomax System						
*ν* = 3	1⋅10^3^	**24** **±** **7**	40 ± 30	58 ± 2	278 ± 2	239 ± 1
	4⋅10^3^	**5** **±** **2**	16 ± 7	56 ± 1	259 ± 9	225 ± 1
	1⋅10^4^	**4** **±** **2**	16 ± 6	55 ± 1	246 ± 1	215 ± 1
	4⋅10^4^	**12** **±** **2**	18 ± 4	53 ± 1	229 ± 1	203 ± 1
*ν* = 4	1⋅10^3^	**17** **±** **8**	25 ± 15	43 ± 2	255 ± 1	222 ± 1
	4⋅10^3^	**3** **±** **3**	16 ± 8	43 ± 1	238 ± 1	210 ± 1
	1⋅10^4^	**7** **±** **3**	15 ± 6	41.1 ± 0.5	224 ± 1	199 ± 1
	4⋅10^4^	**17** **±** **2**	21 ± 7	39.4 ± 0.2	208 ± 1	188 ± 1
*ν* = 100	1⋅10^3^	**15** **±** **7**	25 ± 14	20 ± 1	203 ± 1	185 ± 1
	4⋅10^3^	**5** **±** **2**	18 ± 4	21.4 ± 0.7	187 ± 1	173 ± 1
	1⋅10^4^	**9** **±** **2**	21 ± 4	21.1 ± 0.1	174 ± 1	164 ± 1
	4⋅10^4^	23 ± 3	29 ± 4	**20.4** **±** **0.1**	160 ± 1	153 ± 1
GAUSSIAN source/Trivial System						
	1⋅10^3^	24 ± 1	12 ± 2	**6.0** **±** **0.2**	91.5 ± 0.2	95.6 ± 0.1
	4⋅10^3^	16 ± 3	9 ± 3	**6.4** **±** **0.4**	90.4 ± 0.4	95.1 ± 0.2
	1⋅10^4^	10 ± 2	**4** **±** **1**	6.4 ± 0.2	88.5 ± 0.2	93.8 ± 0.2
	4⋅10^4^	7 ± 3	**3** **±** **2**	6.1 ± 0.2	86.3 ± 0.2	92.3 ± 0.2

The results show that whereas the method proposed in [81] shows better behavior for Gaussian sources, the estimator proposed here, Eq. (7), gives better results with heavy-tailed samples and the kind of systems considered in this work.

## Footnotes

1 In the context of the noisy sensory system defined by Eq. (3), the transmitted information is $I(\boldsymbol{r},\boldsymbol{x}) = h(\boldsymbol{x}) - h( \boldsymbol{x}|\boldsymbol{r})$, so, as the uncertainty of the response given the input is due to the noise, we have $I(\boldsymbol{r},\boldsymbol{x}) = h(\boldsymbol{x}) - h( \boldsymbol{n})$. Then, as the joint entropy is the sum of marginal entropies minus the shared information by all the components, $h(\boldsymbol{x}) = \sum_{i} h(x_{i}) - T(\boldsymbol{x})$, and by plugging this into the previous equation, we get Eq. (8).

## Abbreviations

LMS cones: cones tuned to Long, Medium, and Short wavelength
ATD: Achromatic, Tritanopic, and Deuteranopic color channels
CSF: Contrast Sensitivity Function
PDF: Probability Density Function
CDF: Cumulative Density Function
RBIG: Rotation-Based Iterative Gaussianization
SNR: Signal-to-Noise Ratio
